# Distinct metabolic biomarkers to distinguish IgG4-related disease from Sjogren’s syndrome and pancreatic cancer and predict disease prognosis

**DOI:** 10.1186/s12916-022-02700-x

**Published:** 2022-12-27

**Authors:** Songxin Yan, Yu Peng, Ziyan Wu, Linlin Cheng, Haolong Li, Honglin Xu, Yuan Huang, Wen Zhang, Yongzhe Li

**Affiliations:** 1 Department of Clinical Laboratory, State Key Laboratory of Complex Severe and Rare Diseases, Peking Union Medical College Hospital, Chinese Academy of Medical Science and Peking Union Medical College, Beijing, China; 2 Department of Rheumatology, National Clinical Research Center for Dermatologic and Immunologic Diseases, Key Laboratory of Rheumatology and Clinical Immunology, Ministry of Education, State Key Laboratory of Complex Severe and Rare Diseases, Peking Union Medical College Hospital, Chinese Academy of Medical Science and Peking Union Medical College, Beijing, China

**Keywords:** Immunoglobulin G4-related disease, Metabolome, Diagnosis, Prognosis

## Abstract

**Background:**

The pathogenesis of immunoglobulin G4-related disease (IgG4-RD) remains unclear. IgG4-RD often mimics other diseases, including pancreatic cancer (PC) and Sjogren’s syndrome (SS), which may easily lead to misdiagnosis. This study was performed to explore the metabolite changes and potential biomarkers of IgG4-RD and other misdiagnosed diseases.

**Methods:**

Untargeted liquid chromatography–tandem mass spectrometry metabolomics profiling of plasma samples from a cohort comprising healthy controls (HCs) and patients with IgG4-RD (*n* = 87), PC (*n* = 33), and SS (*n* = 31) was performed. A random forest machine learning model was used to verify the relevance of the identified metabolites in the diagnosis of different diseases and the prediction of disease prognosis.

**Results:**

The ATP-binding cassette transporter pathway was found to be most closely related to IgG4-RD, which was significantly up-regulated in the IgG4-RD group than in all the matched groups. Five metabolites were proved to be valuable biomarkers for IgG4-RD. Caftaric acid, maltotetraose, d-glutamic acid, 1-stearoyl-2-arachidonoyl-sn-glycero-3-phosphoserine, and hydroxyproline were useful in distinguishing between IgG4-RD, PC, SS, and HC [area under the curve (AUC) = 1]. A combination of phenylalanine betaine, 1-(1z-hexadecenyl)-sn-glycero-3-phosphocholine, Pi 40:8, uracil, and N1-methyl-2-pyridone-5-carboxamide showed a moderate value in predicting relapse in patients with IgG4-RD (AUC = 0.8).

**Conclusions:**

Our findings revealed the metabolite changes of IgG4-RD and provide new insights for deepening our understanding of IgG4-RD despite the lack of validation in external cohorts. Metabolomic biomarkers have significance in the clinical diagnosis and disease prognosis of IgG4-RD.

**Supplementary Information:**

The online version contains supplementary material available at 10.1186/s12916-022-02700-x.

## Background

Immunoglobulin G4-related disease (IgG4-RD) is a complex immune-mediated fibroinflammatory condition of unknown etiology and can affect multiple organs [1, 2]. It often manifests with subacute masses in the affected organs or diffuse organ enlargement, which is often confused with neoplastic disease (e.g., pancreatic cancer [PC]) and autoimmune diseases (e.g., primary sclerosing cholangitis [PSC] and Sjogren’s syndrome [SS]), leading to misdiagnosis. Treatment regime and clinical outcomes vary greatly among these diseases [3–5].

Biomarkers for the diagnosis and differential diagnosis of IgG4-RD are urgently needed. Serum IgG4 [6] and IgE levels [7] are the classical markers for the diagnosis of IgG4-RD, which are also associated with disease activity and relapse. As for the biomarkers in immune cells, cells involved in antibody production, such as plasmablasts [8] and follicular helper T (Tfh) cells [9], especially Tfh2 cells, were found to be significantly elevated in patients with untreated IgG4-RD and would gradually decrease as symptoms improved after treatment. Specific autoantibodies, including anti-galectin-3 and anti-interleukin-1 receptor agonist autoantibodies, have been identified in patients with IgG4-RD [10, 11]. However, some biomarkers are of moderate diagnostic value, and more comprehensive and valuable biomarkers need to be uncovered.

The advances in liquid chromatography–mass spectrometry (LC-MS) have revolutionized untargeted metabolomics [12]. Metabolomics can help researchers uncover the key metabolites and their association with diseases. The application of untargeted metabolomics leads to the discovery of new biomarkers and provides new insights on disease pathogenesis and potential therapeutic targets with applications in precision medicine. Metabolic biomarkers of high differential diagnostic values have been identified for distinguishing pancreatic ductal adenocarcinoma from chronic pancreatitis [13]. Seronegative rheumatoid arthritis and psoriatic arthritis show distinct serum metabolomic and lipidomic signatures [14].

IgG4-RD is gradually being considered as an immune-mediated disease with metabolic disorders. Our previous plasma metabolomics study found some differentially expressed metabolites in patients with IgG4-RD compared with healthy volunteers, some of which have potential diagnostic value [15]. A recent study also reported that the fecal metabolomic profiles between patients with IgG4-related sclerosing cholangitis and those with PSC are significantly different [16]. In the present study, we performed untargeted liquid chromatography–tandem mass spectrometry metabolomics profiling of plasma samples of patients with IgG4-RD, PC, and SS and age- and sex-matched healthy controls (HCs) to further comprehensively uncover the metabolic mechanisms and the potential metabolic biomarkers of IgG4-RD.

## Methods

### Study design and participants

This study was based on a prospective cohort (*registered on* *ClinicalTrails.gov**, NCT01670695; study start date: June 2012*) that was recruited at the Peking Union Medical College Hospital. Patients with untreated IgG4-RD, PC, and SS as well as HCs were enrolled in this study from January 2012 to October 2021. IgG4-RD was diagnosed based on the 2019 American College of Rheumatology/European League Against Rheumatism classification criteria [17]. Patients with other autoimmune diseases, infectious diseases, or malignancies were excluded from this study. SS was diagnosed based on the American–European consensus criteria [18], whereas PC was diagnosed by a pathologist based on histological examination (resection or pancreatic fine needle aspiration specimen). HCs were from a population who self-reported no disease and had no abnormal physical examination results and did not take any medication for the last 6 months. The study was conducted in accordance with the guidelines of the Declaration of Helsinki and approved by the Institutional Review Committee of Peking Union Medical College Hospital (approval number: ZS-3193). Written informed consent was obtained from all participants.

### Sample collection and preparation

Blood samples prior to treatment or tumor resection were collected in 2-mL Vacutainer tubes containing the chelating agent ethylenediaminetetraacetic acid. Specimens were collected on an early morning fast. Each participant was required to fast for at least 8 h prior to sample collection. Samples were then centrifuged for 15 min (1500 g, 4°C). Each aliquot (150 μL) of the plasma sample was stored at −80°C until ultra-high performance liquid chromatography–quadrupole time-of-flight mass spectrometry (UHPLC/Q-TOF-MS). The plasma samples were thawed at 4°C, and 100-μL aliquots were mixed with 400 μL of cold methanol/acetonitrile (1:1, v/v) to remove proteins. The mixture was then centrifuged for 15 min (14,000 g, 4°C). The supernatant was dried in a vacuum centrifuge. The samples were redissolved in 100 μL of acetonitrile/water (1:1, v/v) solvent for LC-MS analysis.

### LC-MS/MS analysis and data processing

Analysis was performed using UHPLC (1290 Infinity LC, Agilent Technologies, Palo Alto, CA, USA) coupled to Q-TOF-MS (TripleTOF 6600, AB Sciex, Framingham, MA, USA). Further details on the LC-MS/MS analysis and data processing can be found in Additional file 1.

### Kyoto Encyclopedia of Genes and Genomes (KEGG) analysis

The enrichment analysis of the KEGG metabolic pathway was applied by the KEGG library. Fisher’s exact test was used to analyze and calculate the significance level of the enrichment pathway by MBROLE version 2.0 (http://csbg.cnb.csic.es/mbrole2/).

### Statistical analysis

Statistical analyses were performed by R V.4.0.2. The original data was initially divided into the training set and test set according to 7:3 (Additional file 2: Fig. S1). After the data is preprocessed by total sum-normalization (R base 4.2.1) and pareto-scaling (Ropls 1.28.2), multidimensional statistical analysis is performed only in the training set, including unsupervised principal component analysis (PCA) analysis and orthogonal partial least-squares discriminant analysis (OPLS-DA). The sevenfold cross-validation and response permutation testing were used to evaluate the robustness of the OPLS-DA model. The variable importance in the projection (VIP) value of each variable in the OPLS-DA model was calculated to assess its contribution to the classification. Student’s *t* test was performed to determine the significance of differences between two groups of independent samples. The resulting *P*-values were adjusted by using the Benjamini–Hochberg false discovery rate (FDR) method for multiple testing. VIP > 1 and *P*-value < 0.05 were used to screen for significantly changed metabolites. Spearman’s correlation analysis was performed to determine the correlation between two variables. A two-sided *α* of < 0.05 was considered to indicate statistical significance. In the random forest analysis, a thousand trees were built using the R package randomForest (version 4.6.14) with 10-fold cross-validation. This was repeated 100 times. Then, in the training set, to reduce the impact of the randomness of the data and reduce the overfitting of the model, a 10-fold cross-validation was used for the training set. After the model was established, the test set was used for verification. For each tree, the classification accuracy of the out-of-bag samples was determined both with and without random permutation of the values of the variable. The prediction accuracy after permutation was subtracted from the prediction accuracy before permutation and averaged over all trees in the forest to obtain the importance scores. The importance scores were used to identify biomarkers and remove non-informative variables.

## Results

### Patients’ distribution and clinical features

A total of 151 patients (87 with IgG4-RD, 33 with PC, and 31 with SS) and 30 age- and sex-matched HCs were included in the study (Additional file 2: Fig. S2A). Additional file 3: Tables S1–3 show the disease distribution and detailed demographic features of each group. Patients with IgG4-RD were further classified into those with IgG4-related sialadenitis or dacryoadenitis (IgG4-RSD, *n* = 31) and those with IgG4-related pancreatitis (IgG4-RP, *n* = 33) according to their organ involvements (Additional file 2: Fig. S2B, Additional file 3: Table S4).

### Distinct metabolomics profiling related to the pathogenesis of IgG4-RD

**Fig. 1:**
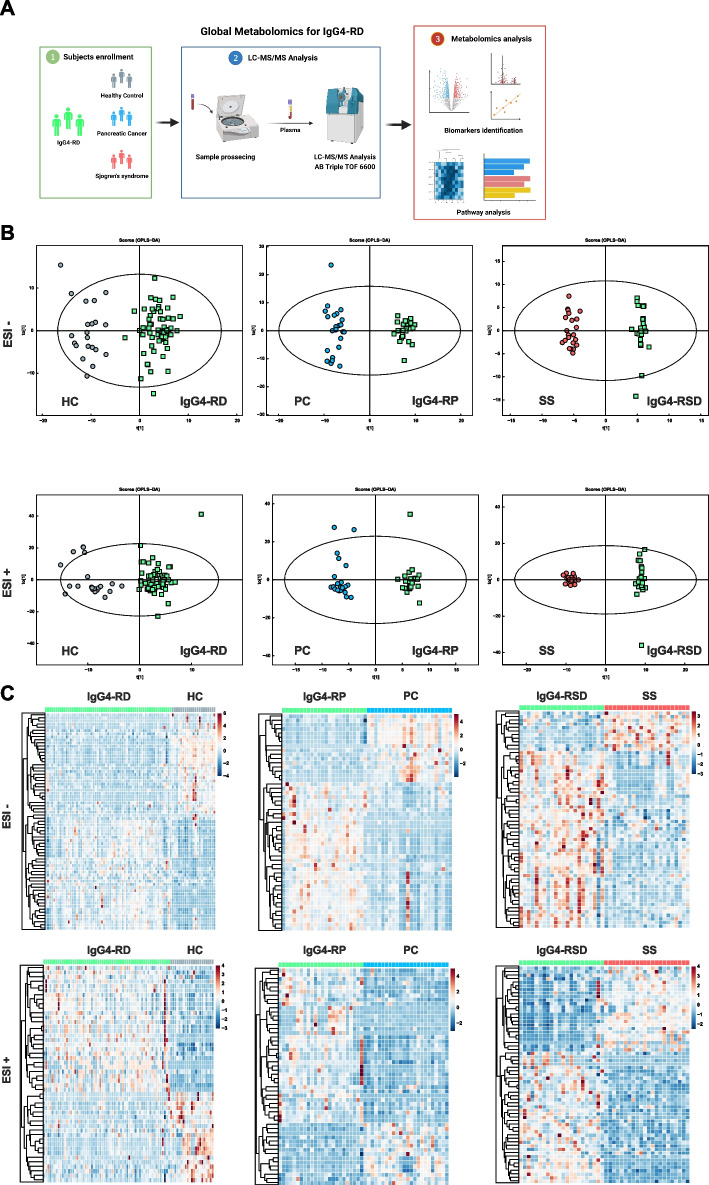
Diverse differently expressed metabolites between IgG4-RD patients and other matched groups. **A** The workflow diagram of this study (created with BioRender.com). **B** ESI+ and ESI− modes of orthogonal partial least-squares discriminant analysis (OPLS-DA) between IgG4-RD patients (including IgG4-RP [*n* = 24] and IgG4-RSD [*n* = 22] patients, blue) and other matched groups (PC, *n* = 24, red; SS, *n* = 22, green; HC, *n* = 21, yellow). **C** ESI+ and ESI− modes of hierarchical clustering between IgG4-RD patients (including IgG4-RP [*n* = 24] and IgG4-RSD [*n* = 22] patients, blue) and other matched groups (PC, *n* = 24, red; SS, *n* = 22, green; HC, *n* = 21, yellow). IgG4-RD, immunoglobulin G4-related disease; HC, healthy control; IgG4-RP, IgG4-related pancreatitis; IgG4-RSD, IgG4-related sialadenitis or dacryoadenitis; SS, Sjogren’s syndrome; PC, pancreatic cancer

We first performed untargeted metabolomics profiling of all enrolled patients’ plasma samples (Fig. 1A). The metabolomics results were analyzed via group comparison, including IgG4-RD group vs. HC group, IgG4-RP group vs. PC group, and IgG4-RSD group vs. SS group. Diverse metabolites were found between different comparison groups (Additional file 2: Fig. S3). PCA and OPLS-DA were then performed to characterize the differential profiling between matched groups. Figure 1B and Additional file 2: Fig. S4 display the positive mode (ESI+) and negative mode (ESI−) of ESI. Different groups could be easily separated via OPLS-DA and PCA. Multiple correlation coefficient (R2) and cross-validated R2 (Q2) in cross-validation and permutation test showed the reliability of the model (Additional file 2: Fig. S5). R2 and Q2 of the model in ESI+ mode were 0.861 and 0.780 (IgG4-RD group vs. HC group), 0.977 and 0.861 (IgG4-RP group vs. PC group), and 0.993 and 0.940 (IgG4-RSD group vs. SS group). R2 and Q2 of the model in ESI− mode were 0.891 and 0.795 (IgG4-RD group vs. HC group), 0.976 and 0.772 (IgG4-RP group vs. PC group), and 0.987 and 0.633 (IgG4-RSD group vs. SS group).

Metabolites with VIP > 1 and FDR < 0.05 were considered to have a significant difference. There were 118 significantly different metabolites between the IgG4-RD group and HC group, of which 64 metabolites were up-regulated in the IgG4-RD group and 54 metabolites were down-regulated in the IgG4-RD group. Among them, caffeic acid, 3-hydroxyoctanoic acid, and vitamin C were the top three metabolites with the most significant FDR, which were low expressed in the IgG4-RD group. The FDR of d-glutamic acid was most significant among highly expressed metabolites in IgG4-RD patients (Additional file 3: Table S5). One hundred four significantly different metabolites were found between the IgG4-RP group and PC group, of which 73 metabolites were up-regulated in the IgG4-RP group and 31 metabolites were down-regulated. The top three significant difference was found in the expression of d-glutamic acid, citrate, and caftaric acid (Additional file 3: Table S6). Between the IgG4-RSD group and SS group, 87 metabolites were up-regulated in the IgG4-RSD group, and 35 metabolites were down-regulated in the IgG4-RSD group. Hydroxyproline was found to be highly expressed in SS patients, with the most significant FDR (Additional file 3: Table S7). Hierarchical clustering of the significantly different metabolites is shown in Fig. 1C. Differentially expressed metabolites showed a distinct metabolomics pattern in the IgG4-RD group compared with the PC group and SS group.

**Fig. 2:**
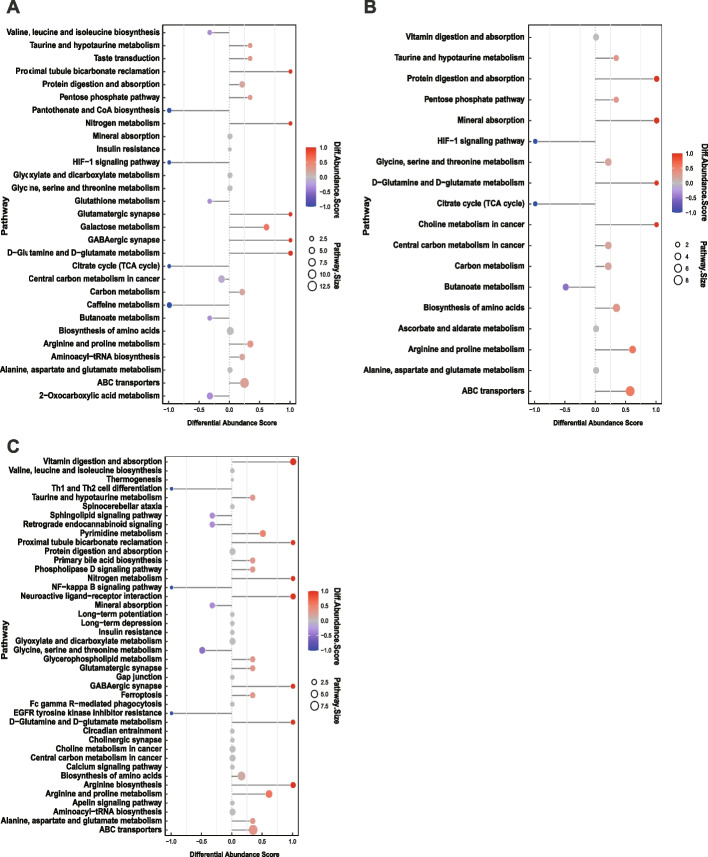
KEGG analysis of the differentially expressed metabolites between IgG4-RD and other matched groups. **A** IgG4-RD (*n* = 61) vs HC (*n* = 21). **B** IgG4-RP (*n* = 24) vs PC (*n* = 24). **C** IgG4-RSD (*n* = 22) vs SS (*n* = 22). DA score represented for the total change of metabolites in the metabolic pathway. A score of 0–1 indicates that all identified metabolites in the pathway have a trend of up-regulation, and −1–0 has a trend of down-regulation of all identified metabolites in this pathway. The length of the line segment represents the absolute value of the DA score. The size of the dot at the endpoint of the line segment represents the number of metabolites in the pathway. The depth of the line segment and dot color is proportional to the DA score value. The darker the red, the more likely the overall expression of the pathway is to be up-regulated, and the darker the blue, the more likely the overall expression of the pathway is down-regulated. IgG4-RD, immunoglobulin G4-related disease; HC, healthy control; IgG4-RP, IgG4-related pancreatitis; IgG4-RSD, IgG4-related sialadenitis or dacryoadenitis; SS, Sjogren’s syndrome; PC, pancreatic cancer

KEGG analysis was further performed to explore the potential metabolic pathways in the pathogenesis of IgG4-RD, and this was compared with other diseases or HCs. Diverse metabolic disorders and pathways were found in this stage. The ATP-binding cassette (ABC) transporter pathway was found to be most closely related to the pathogenesis of IgG4-RD, which was up-regulated in the IgG4-RD group. Thirteen differential metabolites were involved in this pathway, including uridine, taurine, d-lactose, l-valine, d-fructose, glutamic acid, l-glutamine, inosine, hydroxyproline, l-isoleucine, betaine, d-mannose, and leucine (Fig. 2A–C). Additionally, different metabolites were more enriched in the biosynthesis and metabolism of amino acids and some glycolysis-related pathways (HIF-1 signaling pathways), indicating that different metabolic mechanisms might be involved in the development of diseases with similar symptoms.

### Metabolic biomarkers of high diagnostic value in distinguishing IgG4-RD

**Fig. 3:**
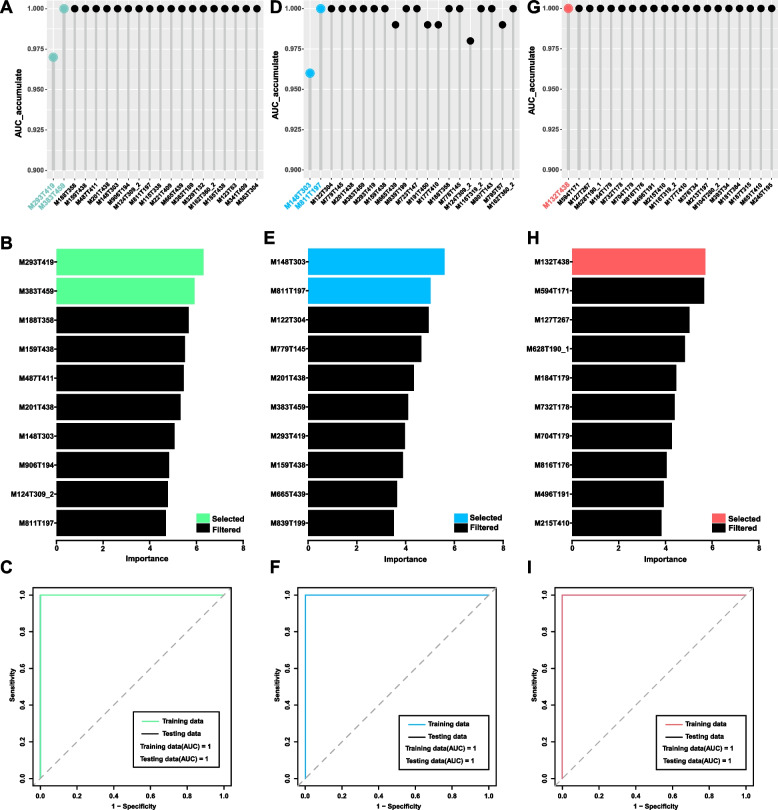
Discovery of the metabolic biomarkers for IgG4-RD. **A**–**C** Metabolic biomarkers for IgG4-RD (*n* = 61) vs HCs (*n* = 21): **A** the cumulative AUC of the differently expressed metabolites between IgG4-RD and HCs; **B** the top 10 metabolites in importance score between IgG4-RD and HCs; **C** ROC curve of the selected metabolites between IgG4-RD and HCs. M293T419, caftaric acid; M383T459, maltotetraose; M188T358, l-glutamine; M159T438, 3-hydroxyoctanoic acid; M487T411, blood group b trisaccharide; M201T438, 1,6-anhydro-2,3-o-isopropylidene-.beta.-d-mannopyranose; M148T303, d-glutamic acid; M906T194, Pi 40:8; M124T309_2, taurine; M811T197, 1-stearoyl-2-arachidonoyl-sn-glycero-3-phosphoserine; M115T338, vitamin c; M221T409, isomaltose; M665T439, fenoxaprop; M362T189, cefadroxil; M329T132, 11beta-hydroxyprogesterone; M162T360_2, carnitine; M185T438, pro-Ala; M123T63, niacinamide; M341T409, d-lactose; M363T304, daunorubicin. **D**–**F** Metabolic biomarkers for IgG4-RP (*n* = 24) vs PC (*n* = 24): **D** the cumulative AUC of the differently expressed metabolites between IgG4-RD and PC; **E** the top 10 metabolites in importance score between IgG4-RP and PC; **F** ROC curve of the selected metabolites between IgG4-RD and PC. M148T303, d-glutamic acid; M811T197, 1-stearoyl-2-arachidonoyl-sn-glycero-3-phosphoserine; M122T304, tris(hydroxymethyl)aminomethane; M779T145, 1-stearoyl-2-myristoyl-sn-glycero-3-phosphocholine; M201T438, 1,6-anhydro-2,3-o-isopropylidene-.beta.-d-mannopyranose; M383T459, maltotetraose; M293T419, caftaric acid; M159T438, 3-hydroxyoctanoic acid; M665T439, fenoxaprop; M839T199, Ps 40:4; M723T147, 1-palmitoyl-2-lauroyl-sn-glycero-3-phosphorylcholine; M191T450, citrate; M177T410, N.omega.-hydroxy-nor-l-arginine; M188T358, l-glutamine; M778T145, 1,2-distearoyl-sn-glycero-3-phospho-(1′-rac-glycerol); M124T309_2, taurine; M116T319_2, l-hydroxyarginine; M807T143, 1-stearoyl-2-palmitoyl-sn-glycero-3-phosphocholine; M795T57, 1-oleoyl-2-palmitoyl-sn-glycero-3-phosphocholine; M162T360_2, Carnitine. **G**–**I** Metabolic biomarkers for IgG4-RSD (*n* = 22) vs SS (*n* = 22): **G** the cumulative AUC of the differently expressed metabolites between IgG4-RSD and SS; **H** the top 10 metabolites in importance score between IgG4-RSD and SS; **I** ROC curve of the selected metabolites between IgG4-RSD and SS. M132T438, hydroxyproline; M594T171, 2-(5-oxovaleryl)phosphatidylcholine; M127T267, imidazoleacetic acid; M628T190_1, 1-stearoyl-2-arachidonoyl-sn-glycerol; M184T179, 1,2-dilauroyl-sn-glycero-3-phosphatidylcholine; M732T178, N-(octadecanoyl)sphing-4-enine-1-phosphocholine; M704T179, palmitoyl sphingomyelin; M816T176, N-tetracosanoyl-4-sphingenyl-1-o-phosphorylcholine; M496T191, Lpc 16:0; M215T410, (+)-6-aminopenicillanic acid; M116T319_2, l-hydroxyarginine; M177T410, N.omega.-hydroxy-nor-l-arginine; M376T34, tuberostemonine; M213T197, m-chlorohippuric acid; M393T34, chenodeoxycholate; M104T280_2, glycerophosphocholine; M181T384, 3,4-dihydroxyhydrocinnamic acid; M651T410, Caylin-1; M187T315, N-acetylglutamine; M245T195, N-acetyltryptophan; IgG4-RD, immunoglobulin G4-related disease; HC, healthy control; IgG4-RP, IgG4-related pancreatitis; IgG4-RSD, IgG4-related sialadenitis or dacryoadenitis; SS, Sjogren’s syndrome; PC, pancreatic cancer

After revealing the distinct metabolomics profile of IgG4-RD, we further explored whether there were metabolic biomarkers to distinguish IgG4-RD from other diseases or HCs. A random forest machine learning model based on metabolomics data and receiver operating characteristic (ROC) analysis was used to verify the relevance of the identified metabolites in the differential diagnosis of IgG4-RD, HC, SS, and PC. The importance score of each metabolite was calculated by random forest, and sorted metabolites (according to their importance score, Fig. 3A, D, G) were used to cumulatively generate ROC curves. Caftaric acid and maltotetraose had a high diagnostic value in distinguishing IgG4-RD and HC [area under the curve (AUC) =1, Fig. 3B, C]. Combining d-glutamic acid and 1-stearoyl-2-arachidonoyl-sn-glycero-3-phosphoserine can adequately distinguish IgG4-RP from PC (AUC = 1, Fig. 3E, F). Hydroxyproline was valuable in distinguishing IgG4-RSD from SS (AUC = 1, Fig. 3H, I).

**Fig. 4:**
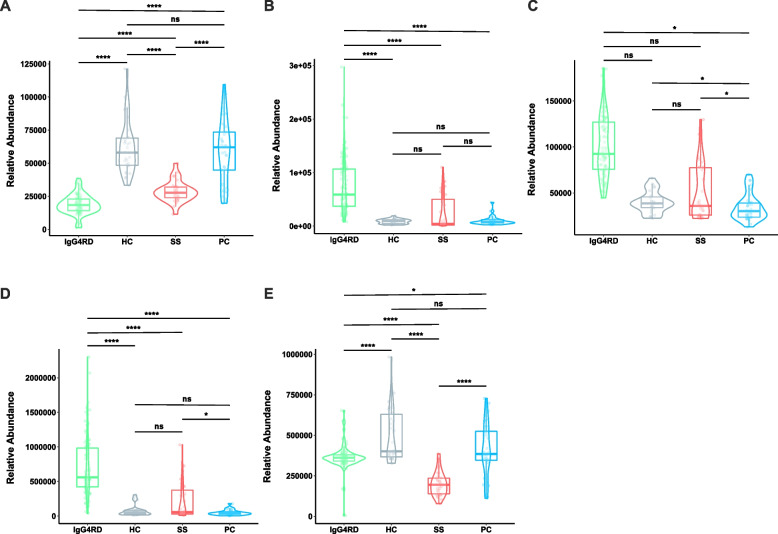
The expression level of selected metabolic biomarkers. **A** Caftaric acid. IgG4-RD (*n* = 87) vs HC (*n* = 30). **B** Maltotetraose. IgG4-RD (*n* = 87) vs HC (*n* = 30). **C**d-Glutamic acid. IgG4-RP (*n* = 33) vs PC (*n* = 33). **D** 1-Stearoyl-2-arachidonoyl-sn-glycero-3-phosphoserine. IgG4-RP (*n* = 33) vs PC (*n* = 33). **E** Hydroxyproline. IgG4-RSD (*n* = 31) vs SS (*n* = 31). The Mann–Whitney test was used for the comparison of the relative abundance between IgG4-RD patients and other groups. *P < 0.05, **P < 0.01, ***P < 0.001, ns represented for not significant. IgG4-RD, immunoglobulin G4-related disease; HC, healthy control; IgG4-RP, IgG4-related pancreatitis; IgG4-RSD, IgG4-related sialadenitis or dacryoadenitis; SS, Sjogren’s syndrome; PC, pancreatic cancer

The selected metabolic biomarkers in this study were significantly increased or decreased in the IgG4-RD group compared with the other disease groups (Fig. 4A–F, Additional file 2: Fig. S6A–F). Some metabolic biomarkers were associated with the clinical features of the disease. There were potential positive correlations between caftaric acid and the levels of several serum immunoglobulins in patients with IgG4-RD, especially serum IgG, IgG1, and IgG4 levels (Additional file 2: Fig. S7). Also, hydroxyproline was found to be negatively related with ESR and positively related with hemoglobin in patients with IgG4-RD. Maltotetraose and 1-stearoyl-2-arachidonoyl-sn-glycero-3-phosphoserine were positively correlated with platelet count.

### Potential metabolites predict IgG4-RD relapse

**Fig. 5:**
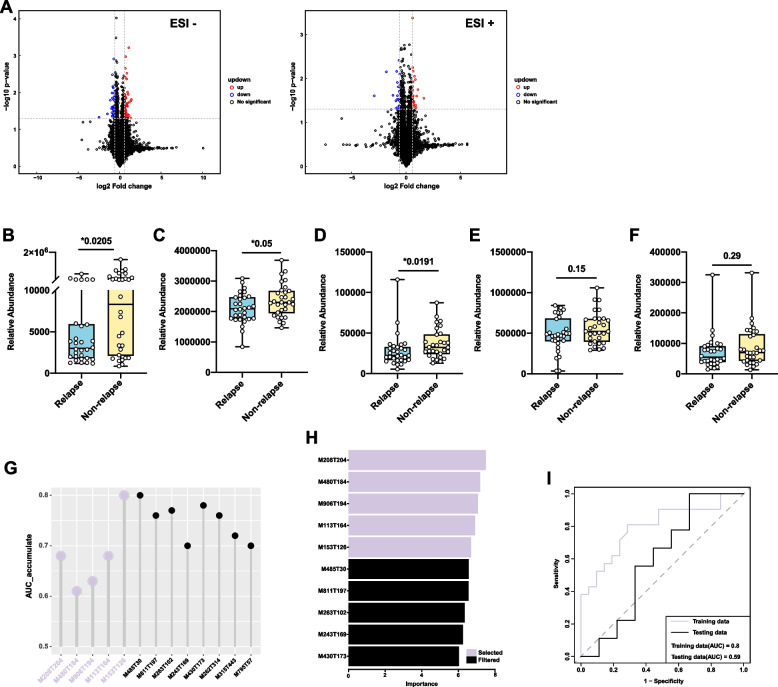
Discovery of the metabolic biomarkers for IgG4-RD relapse. The volcano plot for the differently expressed metabolites between relapsed (*n* = 21) and non-relapsed (*n* = 21) patients in ESI+ and ESI− modes. **B**–**F** The expression levels of the metabolic biomarkers for IgG4-RD relapse (*n* = 30): **B** phenylalanine betaine; **C** 1-(1z-hexadecenyl)-sn-glycero-3-phosphocholine; **D** Pi 40:8; **E** uracil; **F** N1-methyl-2-pyridone-5-carboxamide. The Mann–Whitney test was used for the comparison of the relative abundance between groups. *P < 0.05, **P < 0.01, ***P < 0.001. **G** The cumulative AUC of the differently expressed metabolites between relapsed and non-relapsed IgG4-RD patients. M208T204, phenylalanine betaine; M480T184, 1-(1z-hexadecenyl)-sn-glycero-3-phosphocholine; M906T194, Pi 40:8; M113T164, uracil; M153T126, N1-methyl-2-pyridone-5-carboxamide; M485T30, andrastin a; M811T197, 1-stearoyl-2-arachidonoyl-sn-glycero-3-phosphoserine; M263T102, (+)-abscisic acid; M243T169, uridine; M430T173, (e,2s,3r,4r,5s)-4-acetyloxy-2-amino-3,5,14-trihydroxyicos-6-enoic acid. **H** The importance scores of each differently expressed metabolite and selected metabolite between relapsed and non-relapsed IgG4-RD patients. **I** ROC curve of the selected metabolites between relapsed and non-relapsed IgG4-RD patients. IgG4-RD, immunoglobulin G4-related disease

It has been reported that 24–63% of patients with IgG4-RD often suffer from disease relapse during the follow-up period [19]. Metabolites and metabolic mechanisms might be involved in the relapse of IgG4-RD. To explore the potential biomarkers related to disease relapse, we classified the enrolled patients with IgG4-RD into relapsed (*n* = 30) and non-relapsed (*n* = 30) groups (1:1 matched by sex, age, affected organs, and serum IgG4 levels through propensity score matching [PSM] analysis) according to their prognosis after treatment (Additional file 3: Table S8). Relapse was defined as the new recurrence or worsening of symptoms or imaging findings, with or without re-elevation in serum IgG4 concentrations [19]. Re-elevation in serological IgG4 levels alone was not considered to define a relapse. Multiple differently expressed metabolites were found between the two groups (Fig. 5A). Thirteen significantly different metabolites were found between the relapsed group and non-relapsed group, of which 2 metabolites were up-regulated in the relapsed group and 11 metabolites were down-regulated in the relapsed group (VIP > 1, *P* < 0.05). Random forest analysis revealed that the combination of phenylalanine betaine (Fig. 5B), 1-(1z-hexadecenyl)-sn-glycero-3-phosphocholine (Fig. 5C), Pi 40:8 (Fig. 5D), uracil (Fig. 5E), and N1-methyl-2-pyridone-5-carboxamide (Fig. 5F) has a moderate value in distinguishing between relapsed and non-relapsed patients (AUC = 0.8, Fig. 5G–I).

## Discussion

The similarities in the manifestations of IgG4-RD and other autoimmune diseases or neoplastic disease make the diagnosis or differential diagnoses challenging using existing imaging or laboratory examinations. Several biomarkers in the serum and immune cells for IgG4-RD have been identified, but their diagnostic values are limited. As IgG4-RD is gradually being recognized as a metabolism-related disease [15, 16], we conducted the metabolomics profiling of plasma samples to uncover the potential metabolic biomarkers for the diagnosis of IgG4-RD and its underlying mechanisms. To the best of our knowledge, this is the first study in which metabolomics was used to identify potential biomarkers for the differential diagnosis of IgG4-RD and other confusing diseases.

Consistent with previous studies [15, 16], our metabolomics profiling found several differently expressed metabolites between HCs and other disease controls (PC and SS), suggesting the presence of distinct metabolic patterns and disorders in patients with IgG4-RD. Five metabolites were found to be promising biomarkers for the diagnosis of IgG4-RD. Caftaric acid was identified as a new diagnostic marker for IgG4-RD, which was significantly decreased in patients with IgG4-RD compared with HCs. Caftaric acid was previously reported to have anti-inflammatory effects, which could downregulate the expression of TLR-2 and HLA-DR in monocytes as well as inhibit the production of proinflammatory cytokines [20]. Gut microbiota-derived caffeic acid can modulate the balance of T helper 17 cells and regulatory T cells and thus reduce inflammation [21]. The decrease in caffeic acid might be related to the increase in CD4+CD25+ regulatory T cells in patients with IgG4-RD [22], indicating a potential pathogenesis and treatment target. Maltotetraose is a normal human oligo saccharide and it was elevated in cases of Pompe disease [23]. Our study demonstrated a significant elevation of maltotetraose in patients with IgG4-RD compared with HCs. However, the involvement of maltotetraose in the pathogenesis of IgG4-RD is still unknown.

The imaging findings and serum IgG4 levels of patients with IgG4-RD and pancreatitis often overlap with those of patients with PC [24, 25]. In our study, IgG4-RP could be clearly distinguished from PC using the metabolites d-glutamic acid and 1-stearoyl-2-arachidonoyl-sn-glycero-3-phosphoserine (AUC = 1). Also, d-glutamic acid, which is not endogenously produced in higher mammals and is derived from food intake in humans, was found to be the most potent natural inhibitor of glutathione (GSH) synthesis [26], which is involved in T cell metabolism for inflammation [27]. Specific inhibition of GSH production in mouse T cells could prevent the onset of autoimmune disease. Another metabolic biomarker, i.e., 1-stearoyl-2-myristoyl-sn-glycero-3-phosphocholine, also called PS(18:0/20:4(5Z,8Z,11Z,14Z)), is a glycerophospholipid in which a phosphorylserine moiety occupies a glycerol substitution site and the exposure of phosphorylserine on the outside surface of cells is believed to play a key role in the removal of apoptotic cells [28]. Metabolic biomarker signatures have been previously reported to distinguish PC from chronic pancreatitis with a high diagnostic value [29]. However, no study has investigated the metabolic profile biomarkers between IgG4-RD and PC.

As an immune-mediated disease, IgG4-RD often mimics the symptoms of other autoimmune diseases. To distinguish them, we chose SS as the control disease in this study. Metabolomics revealed that IgG4-RSD had a metabolic pattern distinct from that of SS, and hydroxyproline can adequately distinguish IgG4-RD from SS. Hydroxyproline is a nonessential amino acid mainly found in collagen. It is significantly increased during the fibrosis process of systemic sclerosis [30], which indicate the fibrotic processes involved in the different injury patterns between the two diseases.

Our study also found five metabolic biomarkers (phenylalanine betaine, 1-(1z-hexadecenyl)-sn-glycero-3-phosphocholine, Pi 40:8, uracil, and N1-methyl-2-pyridone-5-carboxamide) that can predict the relapse of IgG4-RD by comparing the metabolomics of relapsed and non-relapsed patients (AUC = 0.8). Few articles have been published on these metabolites. Phenylalanine betaine is an amino acid betaine derived from phenylalanine and it has been reported to significantly increase urinary excretion in mice fed rye brans [31]. 1-(1Z-hexadecenyl)-sn-glycero-3-phosphocholine is a lysophosphatidylcholine P-16:0 with the alk-1-enyl group of hexadec-1-en-1-yl, and lysophosphatidylcholine can inhibit TLR-mediated signaling in the presence of classical TLR ligands, thereby acting as anti-inflammatory molecules [32]. Pi 40:8 is a phosphatidylinositol. Uracil is a common and naturally occurring pyrimidine nucleobase, which is one of the notable pharmacophores in medicinal chemistry as the widespread use of pyrimidine nucleobase in many commercial drugs [33]. N1-methyl-2-pyridone-5-carboxamide is one of the end products of nicotinamide-adenine dinucleotide degradation. A significant increase in the serum concentration of N1-methyl-2-pyridone-5-carboxamide has been observed in chronic renal failure patients, along with the deterioration of kidney function and its toxic properties [34]. These metabolites provide new insights on the potential metabolic mechanisms promoting disease relapse and new treatment targets and regimes to keep the disease stable.

KEGG analysis revealed that the metabolic disorders in IgG4-RD were mainly related to amino acid metabolism and glycolysis pathways. In these pathways, ABC transporters were the most significantly enriched. ABC transporters are a family of membrane proteins found in all domains of life. They contain a pair of ABC or nucleotide-binding domains [35]. Multiple members of ABC transporters were highly expressed on diverse immune cells and closely related to their functions, including the migration of dendritic cells, release of proinflammatory mediators by monocytes and macrophages, polarization of T cells, and differentiation of B cells into memory cells or plasma cells [36]. Notably, some disease-modifying antirheumatic drugs (DMARDs), such as methotrexate, leflunomide, chloroquine, and sulfasalazine, were transported by some ABC transporter members [37, 38]. The expression of these transporters on active immune effector cells was thought to be associated with resistance to these corresponding treatments in autoimmune diseases [39]. Such DMARDs were also widely used in patients with IgG4-RD. Our results indicate the requirement of a change in the treatment of patients with IgG4-RD according to the expression levels of their transporters on the effector cells. However, there were still some limitations in this study. Although we used the permutation test analysis, there is still the possibility of overfitting in the findings. Due to the small number of male patients with SS, we were unable to match the gender ratios of the SS group to the IgG-RSD group, which might have a potential impact on the result. Several limitations should be acknowledged. There was no sample size calculation, and the sample sizes were also limited. This study lacked independent validation in an external cohort, since IgG4-RD is a rare disease. A multi-center verification metabolomics study with a larger sample was also needed.

## Conclusions

In summary, distinct metabolic patterns were observed in patients with IgG4-RD. Five valuable metabolic biomarkers were identified for the diagnosis of IgG4-RD and distinguishing it from PC and SS. Additionally, we found five metabolic biomarkers with moderate values for predicting disease relapse. Amino acid metabolism and glycolysis pathways, especially ABC transporters, have potential roles in the pathogenesis and treatment of IgG4-RD. Despite the limitations mentioned above, we believe that our study further comprehensively uncovered the metabolic mechanisms and the metabolic biomarkers of IgG4-RD.

## Supplementary Information

**Additional file 1.** LC-MS/MS analysis and Data processing.

**Additional file 2: Fig. S1.** Flow diagram of data analysis. **Fig. S2.** The disease distribution of enrolled subjects in this study. **Fig. S3.** Volcano plot representation of the differently expressed metabolites between IgG4-RD and other paired groups. **Fig. S4.** ESI+ and ESI- mode of PCA between groups. **Fig. S5.** Permutation test of OPLS-DA model showing the stability of the model in ESI+ and ESI- mode. **Fig. S6.** The expression level of the selected metabolic biomarkers in all the enrolled subjects. **Fig. S7.** The correlation between metabolic biomarkers and clinical features.

**Additional file 3: Table S1.** Detailed clinical features for the enrolled IgG4-RD patients and healthy controls. **Table S2.** Detailed summary of clinical features for the enrolled Sjogren’s syndrome patients. **Table S3.** Clinical features of pancreatic cancer patients. **Table S4.** Clinical features of IgG4-RSD and IgG4-RP patients. **Table S5.** Differential metabolites between IgG4-RD and HC. Table S6. Differential metabolites between IgG4-RP and PC. **Table S7.** Differential metabolites between IgG4-RSD and SS. **Table S8.** Clinical features of relapsed and non-relapsed IgG4-RD.

## Data Availability

Data are available on reasonable request. All data relevant to the study are included in the article or uploaded as supplementary information. Original LC-MS/MS files are available on reasonable and justified request. Please contact the corresponding author.

## Abbreviations

ABC: ATP-binding cassette
AUC: Area under the curve
DMARDs: Disease-modifying antirheumatic drugs
FDR: False discovery rate
HC: Healthy control
IgG4-RD: Immunoglobulin G4-related disease
IgG4-RP: IgG4-related pancreatitis
IgG4-RSD: IgG4-related sialadenitis or dacryoadenitis
KEGG: Kyoto Encyclopedia of Genes and Genomes
LC-MS: Liquid chromatography–mass spectrometry
OPLS-DA: Orthogonal partial least-squares discriminant analysis
PC: Pancreatic cancer
PCA: Principal component analysis
Q2: Cross-validated R2
R2: Correlation coefficient
ROC: Receiver operating characteristic
SS: Sjogren’s syndrome
Tfh: Follicular helper T
VIP: Variable importance in the projection
