# Supplementary material for: Distinct metabolic biomarkers to distinguish IgG4-related disease from Sjogren’s syndrome and pancreatic cancer and predict disease prognosis
Source: BMC Med. 2022 Dec 27;20:497. doi: 10.1186/s12916-022-02700-x (PMC9795602; doi:10.1186/s12916-022-02700-x)
Supplement: Supplementary file 1 — Additional file 1. LC-MS/MS analysis and Data processing. [file 12916_2022_2700_MOESM1_ESM.docx]

## LC-MS/MS analysis

Analysis was performed using UHPLC (1290 Infinity LC, Agilent Technologies, Palo Alto, CA, USA) coupled to Q-TOF-MS (TripleTOF 6600, AB Sciex, Framingham, MA, USA) in Shanghai Applied Protein Technology Co., Ltd. For hydrophilic interaction liquid chromatography separation, samples were analyzed using a 2.1-mm × 100-mm ACQUIY UPLC BEH 1.7-µm column (Waters, Ireland). In both positive and negative modes of electrospray ionization (ESI), the mobile phase contained A = 25 mM ammonium acetate and 25 mM ammonium hydroxide in water, and B = acetonitrile. The gradient was 85% B for 1 min and was linearly reduced to 65% in 11 min, and then was reduced to 40% in 0.1 min and maintained for 4 min. It was then increased to 85% in 0.1 min, with a re-equilibration period of 5 min.

For reverse-phase liquid chromatography separation, a 2.1-mm × 100-mm ACQUIY UPLC HSS T3 1.8-µm column (Waters) was used. In ESI positive mode, the mobile phase contained A = water with 0.1% formic acid and B = acetonitrile with 0.1% formic acid. In ESI negative mode, the mobile phase contained A = 0.5 mM ammonium fluoride in water and B = acetonitrile. The gradient was 1% B for 1.5 min and was linearly increased to 99% in 11.5 min and maintained for 3.5 min. It was then reduced to 1% in 0.1 min, with a re-equilibration period of 3.4 min. The gradients were at a flow rate of 0.3 mL/min, and the column temperature was kept constant at 25℃. A 2-µL aliquot of each sample was injected.
The ESI source conditions were set as follows: ion source gas 1 (Gas 1) as 60, ion source gas 2

(Gas 2) as 60, curtain gas (CUR) as 30, source temperature as 600℃, and ionspray voltage floating as ±5500 V. In MS only acquisition, the instrument was set to acquire over the m/z range 60–1000 Da, and the accumulation time for TOF MS scan was set at 0.20 s/spectra. In auto MS/MS acquisition, the instrument was set to acquire over the m/z range 25–1000 Da, and the accumulation time for product ion scan was set at 0.05 s/spectra. The product ion scan was acquired using information dependent acquisition under the high sensitivity mode. The parameters were set as follows: the collision energy was fixed at 35 V with ±15 eV, declustering potential (DP), 60 V (+) and −60 V (−), exclude isotopes within 4 Da, and candidate ions to monitor per cycle as 10.

## Data processing

The raw MS data (wiff.scan files) were converted to MzXML files using ProteoWizard MSConvert

before being imported into the freely available XCMS software. The following parameters were used for peak picking: centWave m/z = 25 ppm, peak width = c (10, 60), and prefilter = c (10, 100). For peak grouping, bw = 5, mzwid = 0.025, and minfrac = 0.5 were used. Collection of Algorithms of MEtabolite pRofile Annotation was used for the annotation of isotopes and adducts. In the extracted ion features, the data matrix was processed by removing the peaks with missing values (intensity = 0) in more than 50% of all samples. For the remaining peaks, the missing values were replaced with value of the K Nearest Neighbors (KNN) by R package impute. Compound identification of metabolites was performed by comparing the accuracy of the m/z value (< 25 ppm) and MS/MS spectra with an in-house database established using available authentic standards.
