# Supplementary material for: Distinct metabolic biomarkers to distinguish IgG4-related disease from Sjogren’s syndrome and pancreatic cancer and predict disease prognosis
Source: BMC Med. 2022 Dec 27;20:497. doi: 10.1186/s12916-022-02700-x (PMC9795602; doi:10.1186/s12916-022-02700-x)
Supplement: Supplementary file 2 — Additional file 2: Fig. S1. Flow diagram of data analysis. Fig. S2. The disease distribution of enrolled subjects in this study. Fig. S3. Volcano plot representation of the differently expressed metabolites between IgG4-RD and other paired groups. Fig. S4. ESI+ and ESI- mode of PCA between groups. Fig. S5. Permutation test of OPLS-DA model showing the stability of the model in ESI+ and ESI- mode. Fig. S6. The expression level of the selected metabolic biomarkers in all the enrolled subjects. Fig. S7. The correlation between metabolic biomarkers and clinical features. [file 12916_2022_2700_MOESM2_ESM.docx]

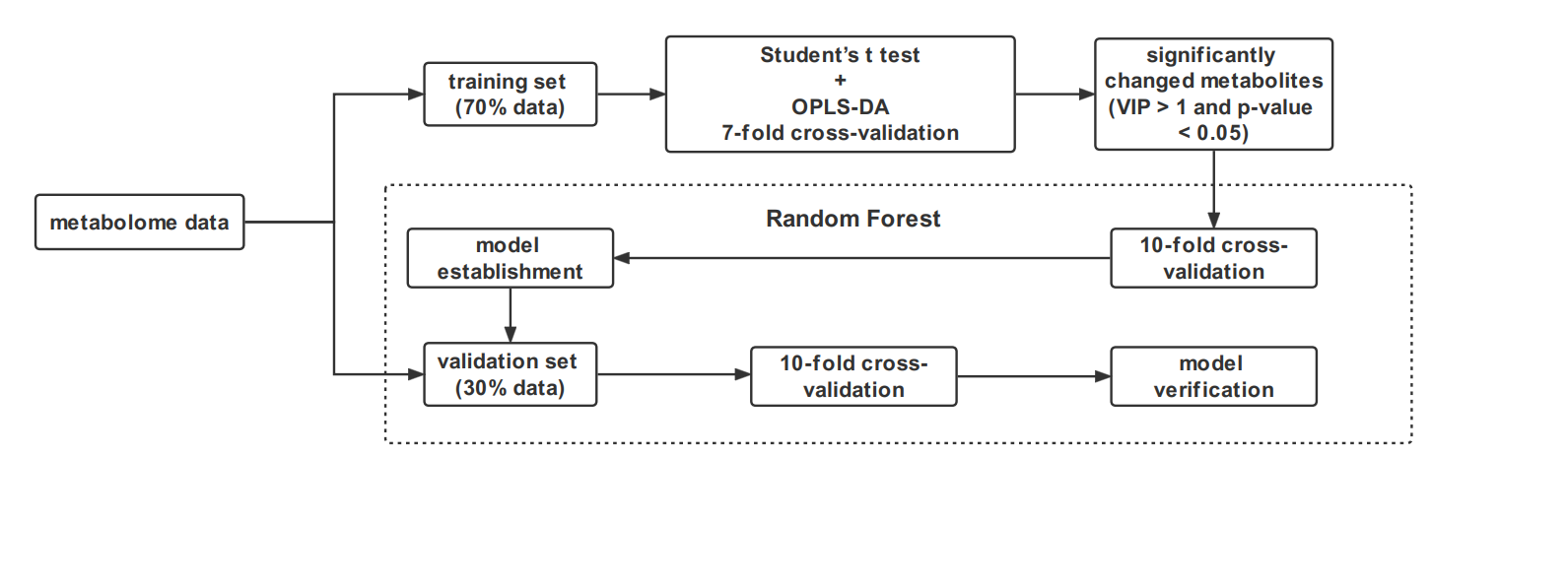


## Fig. S1. Flow diagram of data analysis.


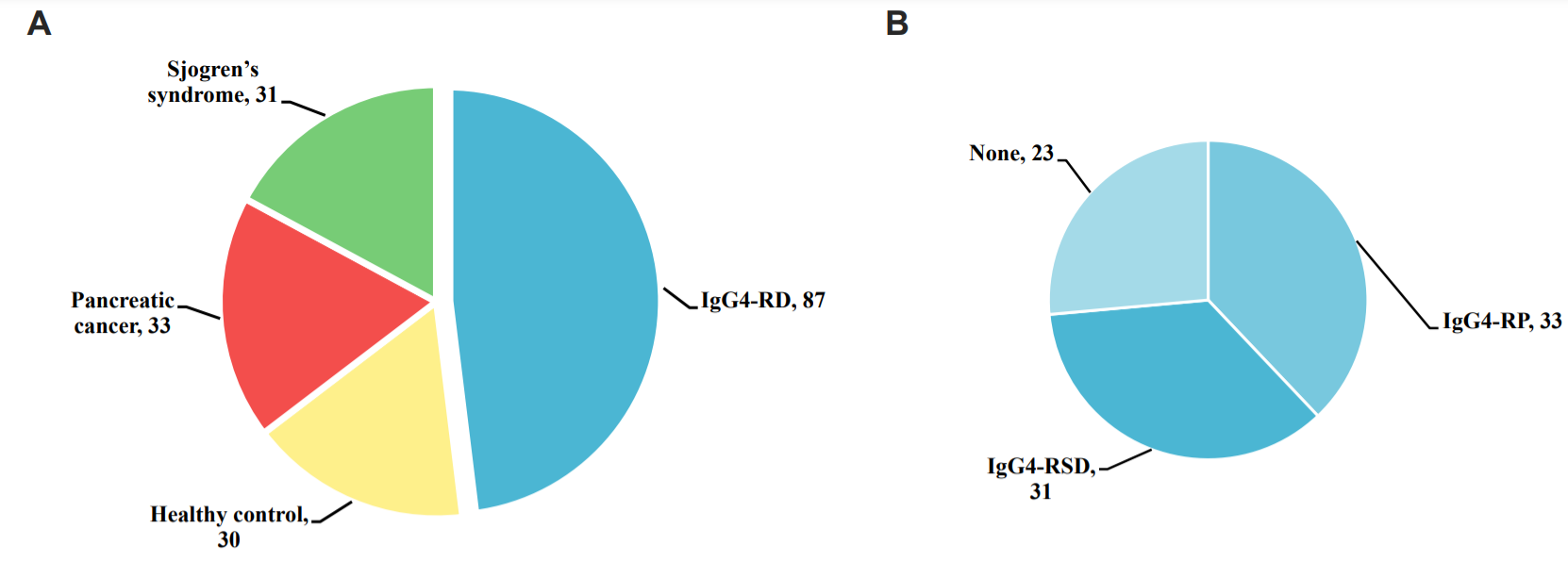


## Fig. S2. The disease distribution of enrolled subjects in this study.

(A) The distribution of all enrolled subjects.

(B) The subclass distribution of the enrolled IgG4-RD patients in this study.

**IgG4-RD, Immunoglobulin G4-related disease; IgG4-RP, IgG4-related pancreatitis; IgG4-RSD, IgG4-related sialadenitis or dacryoadenitis.**


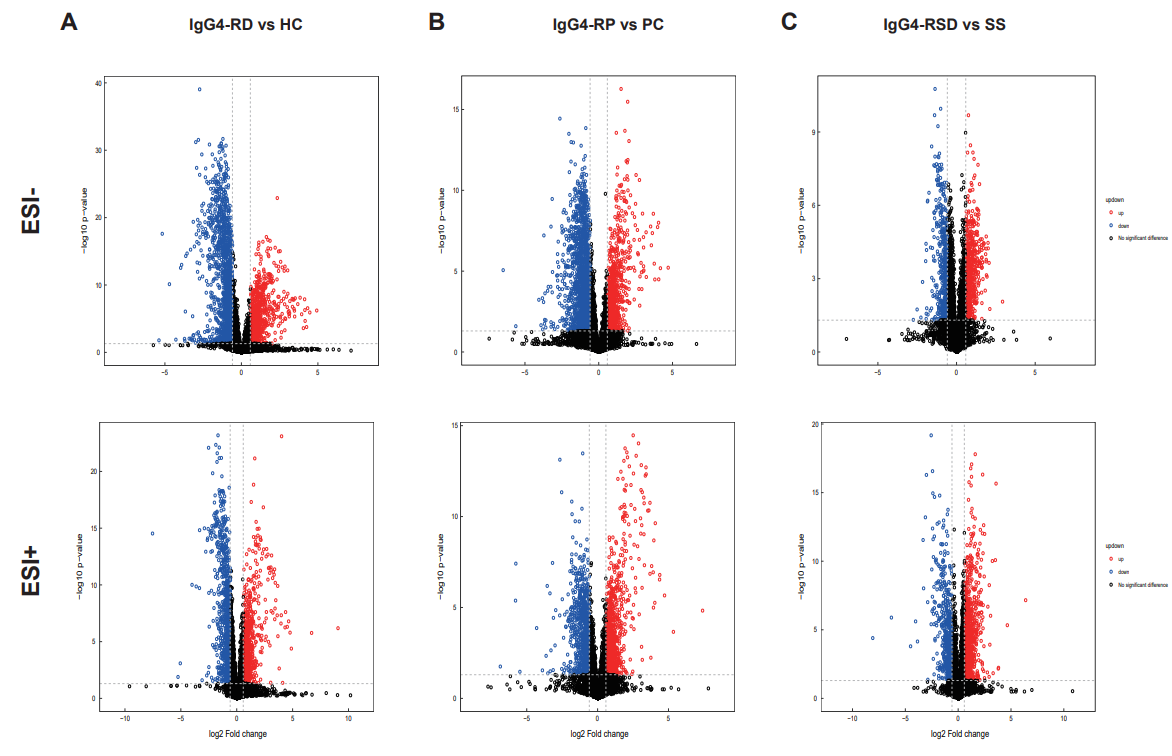


## Fig. S3. Volcano plot representation of the differently expressed metabolites between IgG4-RD and other paired groups (FC >1.5 or FC < 0.67, P < 0.05). Scatter color represents the screening result, red represents significant up-regulation, blue represents significant down-regulation, and black represents non-significant difference metabolites. IgG4-RD, Immunoglobulin G4-related disease; HC, healthy control; IgG4-RP, IgG4-related pancreatitis; IgG4-RSD, IgG4-related sialadenitis or dacryoadenitis; SS, Sjogren’s syndrome; PC, pancreatic cancer.


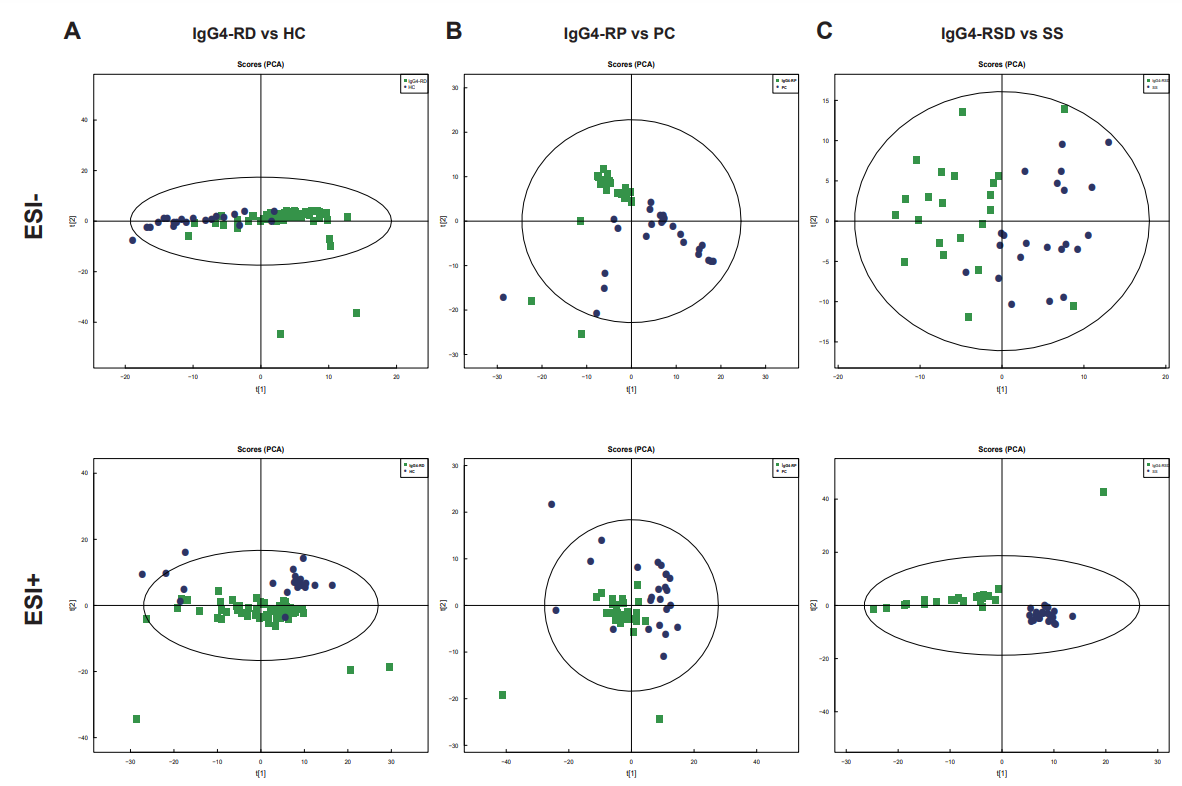


## Fig. S4. ESI+ and ESI- mode of PCA between groups.

**(A) IgG4-RD (n = 61) vs HC (n = 21).**

**(B) IgG4-RP (n = 24) vs PC (n = 24).**

**(C) IgG4-RSD (n = 22) vs SS (n = 22).**

**PCA, Principal Component Analysis; IgG4-RD, Immunoglobulin G4-related disease; HC, healthy control; IgG4-RP, IgG4-related pancreatitis; IgG4-RSD, IgG4-related sialadenitis or dacryoadenitis; SS, Sjogren’s syndrome; PC, pancreatic cancer.**


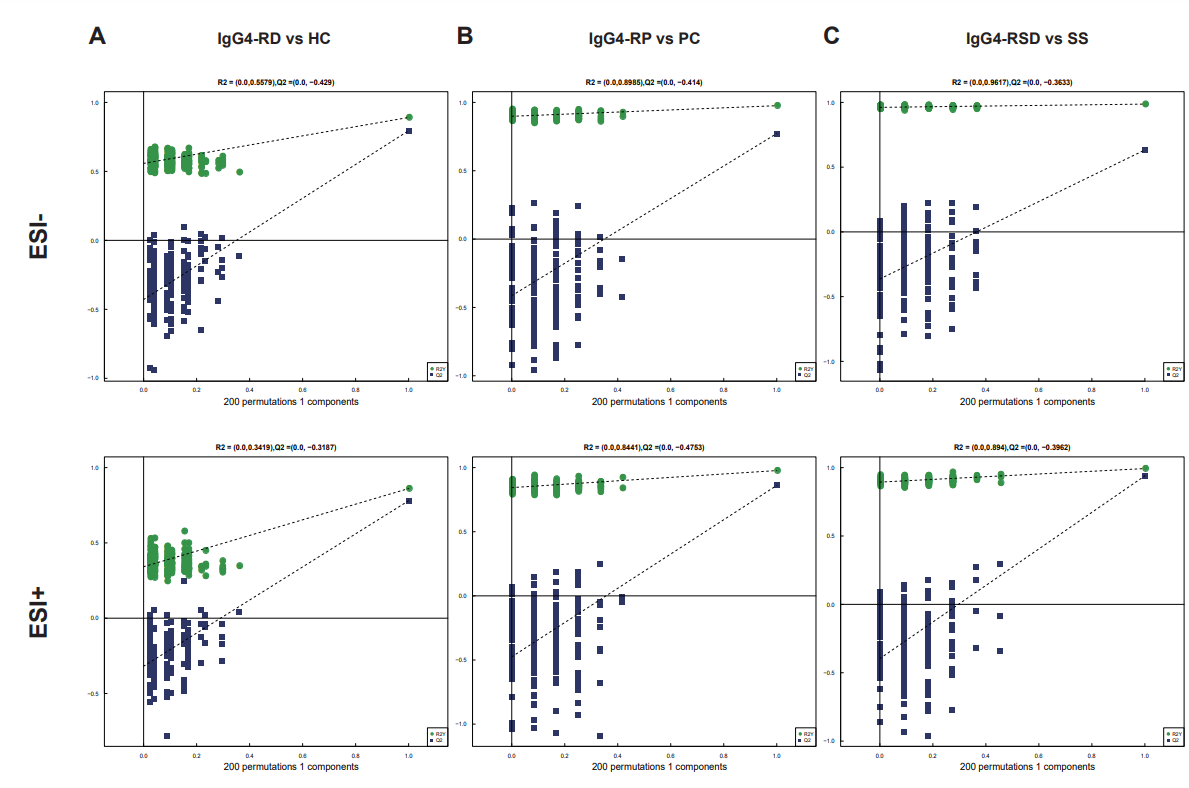


## Fig. S5. Permutation test of OPLS-DA model showing the stability of the model in ESI+ and ESI- mode. The Y-axis shows R2Y and Q2, and the X-axis shows the correlation of observed and permuted data. The green dot indicates the R2Y value, the blue square indicates the Q2 value, and the two dotted lines indicate the regression lines of R2Y and Q2, respectively. The original model R2Y is very close to 1, indicating that the established model conforms to the real situation of the sample data. The original model Q2 is very close to 1, indicating that if a new sample is added to the model, an approximate distribution will be obtained. The numbers in brackets to the right of the R2 and Q2 represent the y-intercept of the regression lines. The Q2 value of the random model of permutation test is smaller than the Q2 value of the original model, the intercept of the regression line of Q2 and the vertical axis is <0, the proportion of the Y variable increases with the decrease in permutation retention, and the Q2 of the stochastic model gradually decreases. This shows that the OPLS-DA model has good robustness and there is no overfitting phenomenon. IgG4-RD, Immunoglobulin G4-related disease; HC, healthy control; IgG4-RP, IgG4-related pancreatitis; IgG4-RSD, IgG4-related sialadenitis or dacryoadenitis; SS, Sjogren’s syndrome; PC, pancreatic cancer.

## (A) IgG4-RD (n = 61) vs HC (n = 21), R2Y = 0.861, Q2 = 0.780 (ESI+), R2Y = 0.891, Q2 = 0.795 (ESI-).

**(B) IgG4-RP (n = 24) vs PC (n = 24), R2Y = 0.977, Q2 = 0.861 (ESI+), R2Y = 0.976, Q2 = 0.772 (ESI-).**

**(C) IgG4-RSD (n = 22) vs SS (n = 22), R2Y = 0.993, Q2 = 0.940 (ESI+), R2Y = 0.987, Q2 = 0.633 (ESI-).**


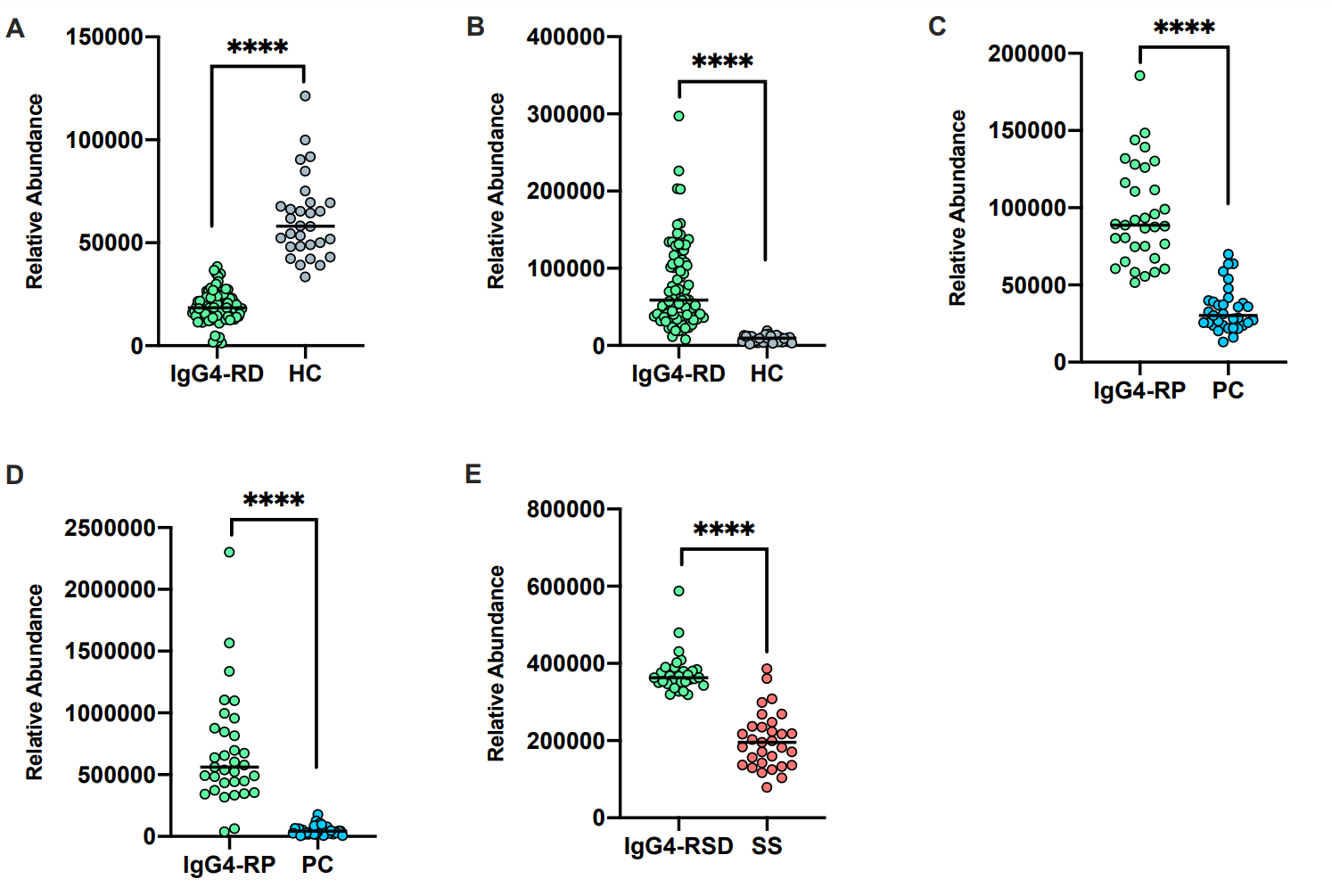


## Fig. S6. The expression level of the selected metabolic biomarkers in all the enrolled subjects.

1. Caftaric acid.
2. Maltotetraose
3. D-Glutamic acid.
4. 1-stearoyl-2-arachidonoyl-sn-glycero-3-phosphoserine.
5. Hydroxyproline.

(Mann-Whitney test was used for the comparison of the relative abundance between groups. *P < 0.05, **P < 0.01, ***P < 0.001.) IgG4-RD, Immunoglobulin G4-related disease; HC, healthy control; IgG4-RP, IgG4-related pancreatitis; IgG4-RSD, IgG4-related sialadenitis or dacryoadenitis; SS, Sjogren’s syndrome; PC, pancreatic cancer.


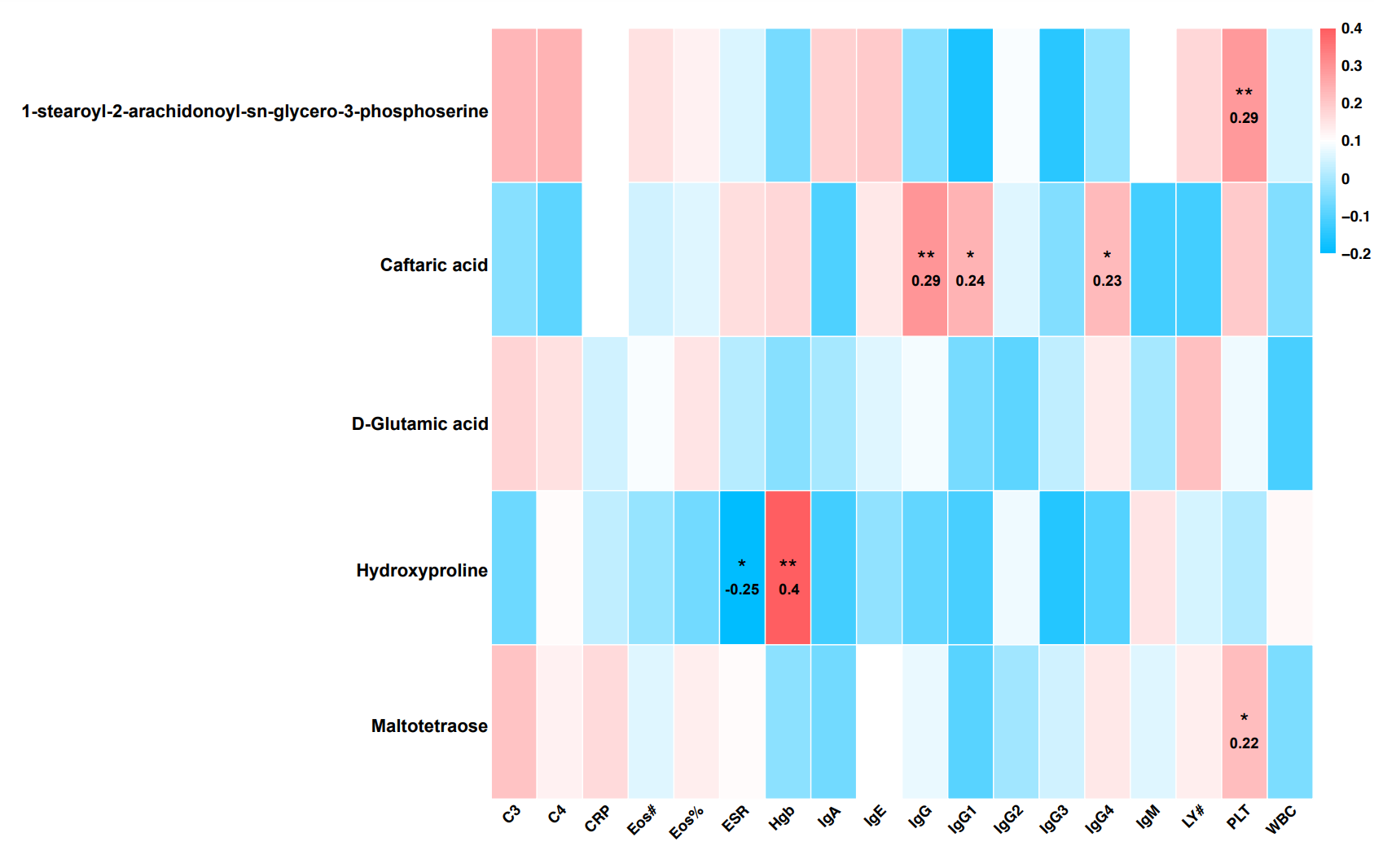


## Fig. S7. The correlation between metabolic biomarkers and clinical features.

(Spearman’s correlation; *P < 0.05, **P < 0.01, ***P < 0.001).
