# Supplementary material for: Distinct metabolic biomarkers to distinguish IgG4-related disease from Sjogren’s syndrome and pancreatic cancer and predict disease prognosis
Source: BMC Med. 2022 Dec 27;20:497. doi: 10.1186/s12916-022-02700-x (PMC9795602; doi:10.1186/s12916-022-02700-x)
Supplement: Supplementary file 3 — Additional file 3: Table S1. Detailed clinical features for the enrolled IgG4-RD patients and healthy controls. Table S2. Detailed summary of clinical features for the enrolled Sjogren’s syndrome patients. Table S3. Clinical features of pancreatic cancer patients. Table S4. Clinical features of IgG4-RSD and IgG4-RP patients. Table S5. Differential metabolites between IgG4-RD and HC. Table S6. Differential metabolites between IgG4-RP and PC. Table S7. Differential metabolites between IgG4-RSD and SS. Table S8. Clinical features of relapsed and non-relapsed IgG4-RD. [file 12916_2022_2700_MOESM3_ESM.docx]

## Table S1. Detailed clinical features for the enrolled IgG4-RD patients and healthy controls.

|  | **IgG4-RD**  **（n=87）** | **HC**  **(n=30)** |
| --- | --- | --- |
| **Demography** |  |  |
| Sex (male，%) | 54 (62.1%) | 20 (66.7%) |
| Age at onset (years, mean ± S.D.) | 54.07±12.07 | 54.90±6.09 |
| Disease duration (months, median, IQR) | 24(6-48) | N.A. |
| Affected organ numbers (mean ± S.D.) | 3.29±1.44 | N.A. |
| **Serology (Median, IQR)** |  |  |
| IgG (g/L) | 18.79 (14.69-25.78) | N.A. |
| IgA (g/L) | 1.82 (1.30-2.41) | N.A. |
| IgM (g/L) | 0.71 (0.56-1.01) | N.A. |
| IgE (KU/L) | 361.00 (144.00-726.00) | N.A. |
| RF (IU/L) | 12.20 (3.40-42.00) | N.A. |
| ESR (mm/h) | 15.50 (8.00-36.00) | N.A. |
| CRP (mg/L) | 1.75 (0.73-4.80) | N.A. |
| C3 (g/L) | 0.92 (0.73-1.08) | N.A. |
| C4 (g/L) | 0.16 (0.12-0.24) | N.A. |
| **Organ involvements (n%)** |  |  |
| Lacrimal gland | 54 (62.1%) | N.A. |
| Parotid gland | 12 (13.8%) | N.A. |
| Submandibular gland | 52 (59.8%) | N.A. |
| Pancreas | 33 (37.9%) | N.A. |
| Bile duct | 16 (18.4%) | N.A. |
| Lung | 21 (24.1%) | N.A. |
| Kidney | 7 (8.0%) | N.A. |
| Retroperitoneum | 10 (11.5%) | N.A. |
| Paranasal sinus | 30 (34.5%) | N.A. |
| Thyroid | 0 | N.A. |
| Pituitary | 1 (1.1%) | N.A. |
| Lymph node | 32 (36.8%) | N.A. |

**Variables are described as mean ± S.D. or median, IQR according to whether they satisfied normal distribution.**

**N.A. represented for Not available.**

**IgG, IgA, IgM, IgE represented for immunoglobulin G, A, M and E. RF represented for rheumatoid factor. ESR represented for erythrocyte sedimentation rate, CRP represented for C-reaction protein.**

## Table S2. Detailed summary of clinical features for the enrolled Sjogren’s syndrome patients.

|  | **SS**  **(n=31)** |
| --- | --- |
| **Demography**  Sex (male, n%) | 1 (3.2%) |
| Age at onset (years, mean ± SD) | 48.26±15.28 |
| Disease duration (months, median, IQR) | 24 (6-51) |
| **Serology (Median, IQR)** |  |
| IgG (g/L) | 19.6 (15.8-26.6) |
| IgA (g/L) | 3.3 (2.3-4) |
| IgM (g/L) | 1.1 (0.7-1.3) |
| RF (IU/L) | 130 (28.5-216.8) |
| ESR (mm/h) | 30 (12-50.3) |
| CRP (mg/L) | 0.7 (0.2-1.6) |
| C3 (g/L) | 1.02 (0.91-1.13) |
| C4 (g/L) | 0.17 (0.16-0.21) |

**Variables are described as mean ± S.D. or median, IQR according to whether they satisfied normal distribution.**

**IgG, IgA, IgM represented for immunoglobulin G, A, M. RF represented for rheumatoid factor. ESR represented for erythrocyte sedimentation rate, CRP represented for C-reaction protein.**

## Table S3. Clinical features of pancreatic cancer patients.

|  | **PC** |
| --- | --- |
|  | **(n=33)** |
| **Demography**  Sex (male, n%) | 22 (66.7%) |
| Age at onset (years, mean ± SD) | 55.52±6.17 |
| CA19-9 | 241.00 (99.80-985.00) |
| **Stage** |  |
| ⅠA | 1 |
| ⅠB | 0 |
| ⅡA | 1 |
| ⅡB | 9 |
| Ⅲ | 8 |
| Ⅳ | 14 |

**The stage of pancreatic cancer was evaluated through The American Joint Committee on Cancer (AJCC) staging manual.**

## Table S4. Clinical features of IgG4-RSD and IgG4-RP patients.

|  | **IgG4-RSD**  **(n=31)** | **IgG4-RP**  **(n=33)** |
| --- | --- | --- |
| **Demography**  Sex (male，%) | 14 (45.2%) | 23 (69.7%) |
| Age at onset (years, mean ± SD) | 52.68±13.33 | 54.39±10.68 |
| Disease duration (months, median, IQR) | 24(8-60) | 12(3-36) |
| Affected organ numbers (mean ± SD) | 2.90±1.25 | 3.84±1.25 |
| IgG4 (g/L) | 11.50 (5.47-17.00) | 12.34 (5.07-23.15) |
| **Organ involvement (n, %)** |  |  |
| Lacrimal gland | 27 (87.1%) | 14 (42.4%) |
| Parotid gland | 8 (25.8%) | 1 (3.0%) |
| Submandibular gland | 22 (71.0%) | 18 (54.5%) |
| Pancreas | 0 | 33 (100.0%) |
| Bile duct | 0 | 15 (45.5%) |
| Lung | 8 (25.8%) | 6 (18.2%) |
| Kidney | 1 (3.2%) | 5 (15.2%) |
| Retroperitoneum | 2 (6.5%) | 4 (12.1%) |
| Paranasal sinus | 14 (45.2%) | 6 (18.2%) |
| Thyroid | 0 | 1 (3.0%) |
| Pituitary | 0 | 0 |
| Lymph node | 8 (25.8%) | 15 (45.5%) |

**Variables are described as mean ± S.D. or median, IQR according to whether they satisfied normal distribution.**

## Table S5. Differential metabolites between IgG4-RD and HC.

| **Name** | **VIP** | **Fold change** | **p-value** | **FDRvalue** |
| --- | --- | --- | --- | --- |
| Caftaric acid | 1.37371 | 0.349864 | 1.05E-25 | 2.03E-23 |
| 3-hydroxyoctanoic acid | 2.491862 | 0.566789 | 3.08E-19 | 1.52E-17 |
| Vitamin c | 2.427177 | 0.503622 | 1.47E-18 | 6.42E-17 |
| 11beta-hydroxyprogesterone | 1.004679 | 0.492976 | 3.81E-18 | 1.56E-16 |
| 1,6-anhydro-2,3-o-isopropylidene-.beta.-d-mannopyranose | 1.947353 | 0.605091 | 1.24E-17 | 4.69E-16 |
| Fenoxaprop | 3.581314 | 0.56123 | 2.59E-16 | 8.08E-15 |
| Pro-Ala | 1.005497 | 0.576286 | 1.02E-15 | 2.92E-14 |
| Citrate | 1.801468 | 0.463826 | 1.94E-13 | 4.3E-12 |
| D-Glutamic acid | 1.131263 | 2.654545 | 1.07E-13 | 6.69E-12 |
| Carnitine | 7.981761 | 1.809679 | 2E-13 | 1.15E-11 |
| Inosine | 1.568216 | 0.295041 | 1.04E-11 | 4.22E-10 |
| Cefadroxil | 1.855211 | 2.827154 | 6.19E-11 | 1.04E-09 |
| 2-hydroxy-6-methylquinoline-3-carbaldehyde | 3.404106 | 0.21533 | 9.7E-11 | 1.59E-09 |
| L-pyroglutamic acid | 6.349948 | 0.542596 | 2.35E-10 | 3.67E-09 |
| Taurine | 10.53449 | 2.735262 | 4.01E-10 | 6.13E-09 |
| Sarcosine | 1.716398 | 1.79803 | 3.86E-10 | 1.12E-08 |
| L-homocitrulline | 1.827723 | 0.498014 | 8.28E-10 | 2.23E-08 |
| Blood group b trisaccharide | 1.171975 | 5.384553 | 5.88E-09 | 7.55E-08 |
| Dl-lactate | 7.720145 | 0.284793 | 7.54E-09 | 9.55E-08 |
| 2-furanpropanoic acid, 3-carboxy-4-methyl-5-propyl- | 3.867178 | 0.256987 | 7.78E-09 | 9.84E-08 |
| Uridine | 3.360661 | 0.641906 | 1.44E-08 | 1.74E-07 |
| 1-stearoyl-2-arachidonoyl-sn-glycero-3-phosphoserine | 5.741846 | 10.62055 | 1.53E-08 | 1.84E-07 |
| N-tetracosanoyl-4-sphingenyl-1-o-phosphorylcholine | 3.616988 | 0.328381 | 2.33E-08 | 4.65E-07 |
| N.omega.-hydroxy-nor-l-arginine | 1.594396 | 0.692397 | 3.17E-08 | 6.14E-07 |
| Lactose | 1.265622 | 3.886928 | 8.83E-08 | 1.56E-06 |
| Maltotetraose | 1.689466 | 9.953293 | 1.77E-07 | 1.81E-06 |
| Niacinamide | 2.960793 | 2.654764 | 1.66E-07 | 2.74E-06 |
| L-methionine | 1.178896 | 0.45328 | 3.61E-07 | 3.53E-06 |
| Caylin-1 | 2.443314 | 0.741655 | 2.34E-07 | 3.72E-06 |
| Guanidinopropionic acid | 3.854962 | 0.679827 | 4.21E-07 | 4.07E-06 |
| 1,3-benzenediol, 4-chloro-6-[5-[[2-(4-morpholinyl)ethyl]amino]-1,2-benzisoxazol-3-yl]- | 1.689646 | 1.766704 | 2.98E-07 | 4.63E-06 |
| N-acetylsphingosine | 1.072117 | 0.309353 | 4.05E-07 | 6.02E-06 |
| Pi 40:8 | 1.061096 | 4.269194 | 7.25E-07 | 6.67E-06 |
| 5-aminovaleric acid betaine | 1.223433 | 0.362592 | 6.1E-07 | 8.66E-06 |
| Pantoprazole | 1.553649 | 523.9026 | 6.65E-07 | 9.34E-06 |
| N-acetyldihydrosphingosine | 1.011945 | 0.380844 | 9.25E-07 | 1.27E-05 |
| Omeprazole sulfone n-oxide | 1.382953 | 101.8473 | 1.73E-06 | 2.18E-05 |
| 2-arachidonoyl-1-palmitoyl-sn-glycero-3-phosphoethanolamine | 6.676373 | 2.105859 | 3.27E-06 | 2.69E-05 |
| D-lactose | 1.092044 | 2.391239 | 3.36E-06 | 2.76E-05 |
| Linoleoylcarnitine | 3.608267 | 0.658722 | 2.74E-06 | 3.29E-05 |
| L-Glutamine | 1.225139 | 3.957426 | 2.74E-06 | 3.29E-05 |
| Pe(16:0e/12-hete) | 5.004591 | 1.86675 | 4.14E-06 | 3.33E-05 |
| Daunorubicin | 1.162907 | 3.714975 | 2.96E-06 | 3.5E-05 |
| Isomaltose | 1.131553 | 11.00632 | 4.91E-06 | 3.9E-05 |
| L-Valine | 2.537561 | 0.550457 | 5.29E-06 | 4.16E-05 |
| N-(octadecanoyl)sphing-4-enine-1-phosphocholine | 6.708357 | 0.477417 | 3.68E-06 | 4.25E-05 |
| Hydroxyproline | 1.639721 | 0.732622 | 3.93E-06 | 4.51E-05 |
| D-gluconate | 1.425341 | 1.958427 | 6.41E-06 | 4.97E-05 |
| 3',5'-cyclic inosine monophosphate | 1.464772 | 0.820884 | 7.68E-06 | 5.84E-05 |
| L-hydroxyarginine | 3.715939 | 1.686603 | 5.37E-06 | 5.98E-05 |
| 1-methylxanthine | 1.856046 | 0.316471 | 8.76E-06 | 6.62E-05 |
| D-erythro-imidazolylglycerol phosphate | 1.186329 | 1.707986 | 8.07E-06 | 8.58E-05 |
| 1-stearoyl-2-myristoyl-sn-glycero-3-phosphocholine | 5.167746 | 1.982662 | 1.91E-05 | 0.000134 |
| S-Methyl-5'-thioadenosine | 1.047865 | 1.888273 | 1.55E-05 | 0.000152 |
| L-Gulonic gamma-lactone | 1.233358 | 1.616461 | 2.23E-05 | 0.000154 |
| .alpha.-L-Glu-L-Asp | 1.124743 | 0.515601 | 2.31E-05 | 0.000159 |
| Pyruvate | 2.900451 | 0.641277 | 2.66E-05 | 0.000181 |
| Hypoxanthine | 3.4569 | 0.354763 | 2.04E-05 | 0.000193 |
| Ser-Thr | 1.701011 | 1.715826 | 3.25E-05 | 0.00029 |
| 2-Amino-2-methyl-1,3-propanediol | 2.597361 | 1.613527 | 3.49E-05 | 0.000309 |
| N-alpha-acetyl-l-lysine | 2.708001 | 1.474125 | 4.28E-05 | 0.00037 |
| D-glucono-1,5-lactone | 1.799719 | 2.825958 | 6.24E-05 | 0.000389 |
| 1-stearoyl-2-palmitoyl-sn-glycero-3-phosphocholine | 1.404971 | 2.023369 | 7.19E-05 | 0.000443 |
| Val-Ala-Lys | 1.60023 | 1.524604 | 5.4E-05 | 0.000447 |
| 2-linoleoyl-1-palmitoyl-sn-glycero-3-phosphoethanolamine | 4.782282 | 2.065002 | 7.65E-05 | 0.000469 |
| 1-o-hexadecyl-2-o-(2e-butenoyl)-sn-glyceryl-3-phosphocholine | 1.977204 | 1.3096 | 7.41E-05 | 0.000591 |
| Betaine | 7.725079 | 0.763159 | 8.14E-05 | 0.00064 |
| Creatine | 2.420812 | 4.641118 | 8.73E-05 | 0.000682 |
| Dihydrothymine | 1.173996 | 0.722111 | 0.000159 | 0.000898 |
| 1-palmitoyl-2-lauroyl-sn-glycero-3-phosphorylcholine | 11.42779 | 1.688338 | 0.000208 | 0.001141 |
| Nicotinuric acid | 1.465058 | 0.400595 | 0.000161 | 0.001156 |
| L-Isoleucine | 2.399903 | 1.539747 | 0.000228 | 0.001558 |
| Tuberostemonine | 5.882012 | 1.581393 | 0.000249 | 0.00168 |
| 1,2-dilauroyl-sn-glycero-3-phosphatidylcholine | 3.474728 | 0.691997 | 0.000346 | 0.002231 |
| Chenodeoxycholate | 2.526976 | 1.616957 | 0.000388 | 0.00246 |
| [6]-gingerol | 1.301689 | 1.281772 | 0.000522 | 0.002603 |
| Pi 38:5 | 7.627879 | 2.128874 | 0.000543 | 0.002695 |
| UDP-D-Galactose | 1.331223 | 0.208139 | 0.000669 | 0.003251 |
| Theophylline | 3.037756 | 0.327578 | 0.000738 | 0.00354 |
| Cis,cis-muconic acid | 12.73457 | 0.82361 | 0.000832 | 0.003932 |
| Lpc 18:2 | 19.5806 | 1.281553 | 0.000857 | 0.00482 |
| Tartronate | 1.357139 | 1.410154 | 0.001085 | 0.004971 |
| Pc 42:10 | 1.964177 | 2.261461 | 0.001173 | 0.005323 |
| D-Mannose | 1.198359 | 1.482934 | 0.001009 | 0.005521 |
| m-Chlorohippuric acid | 2.272069 | 1.463558 | 0.001275 | 0.005715 |
| 5'-O-methylthymidine | 1.174884 | 0.321879 | 0.001498 | 0.0066 |
| D-lyxose | 2.105276 | 0.470268 | 0.001503 | 0.006622 |
| Pi(18:0/8,9-eet) | 2.075538 | 1.768276 | 0.001765 | 0.007638 |
| Sm d34:1 | 1.289312 | 1.317326 | 0.001831 | 0.007886 |
| Leu-Thr-Arg | 2.530192 | 0.320236 | 0.001855 | 0.009235 |
| Pi(16:0e/15-hete) | 3.367606 | 1.927433 | 0.002227 | 0.009352 |
| 1-palmitoyl-2-docosahexaenoyl-sn-glycero-3-phosphocholine | 3.875075 | 0.759985 | 0.002013 | 0.009892 |
| 1,2-dioleoyl-sn-glycero-3-phosphoethanolamine-n,n-dimethyl | 3.503556 | 1.501505 | 0.002452 | 0.010165 |
| Phenylbenzimidazolesulfonic acid | 1.32376 | 0.113022 | 0.00271 | 0.011039 |
| Pi(18:0/9-hode) | 1.205922 | 1.846651 | 0.002835 | 0.011424 |
| Glutamic acid | 1.04051 | 1.981703 | 0.003115 | 0.012404 |
| Pe 38:4 | 13.54808 | 1.559475 | 0.003671 | 0.014333 |
| Ps 40:4 | 1.042435 | 2.020182 | 0.003923 | 0.015108 |
| Asiatic acid | 1.259908 | 0.649269 | 0.004382 | 0.016506 |
| 1,7-Dimethylxanthine | 1.968815 | 0.354196 | 0.005553 | 0.020145 |
| (2-aminoethoxy)[3-[hexadec-1-en-1-yloxy]-2-[icosa-5.8.11.14-tetraenoyloxy]propoxy]phosphinic acid | 4.770704 | 1.476822 | 0.00596 | 0.021369 |
| 1-stearoyl-2-linoleoyl-sn-glycero-3-phosphoethanolamine | 10.3481 | 1.229917 | 0.006744 | 0.023673 |
| Mitoxantrone | 1.100956 | 1.54706 | 0.007264 | 0.025229 |
| Caffeine | 2.354277 | 0.358148 | 0.006378 | 0.025469 |
| 1-Oleoyl-sn-glycero-3-phosphocholine | 12.22872 | 1.264781 | 0.006734 | 0.02657 |
| DL-arginine | 1.261494 | 1.335957 | 0.007251 | 0.028286 |
| Piperine | 2.37349 | 0.390064 | 0.00947 | 0.03529 |
| Beta-octylglucoside | 1.358281 | 1.679035 | 0.010094 | 0.036993 |
| Octanoylcarnitine | 2.396024 | 0.672151 | 0.01052 | 0.03825 |
| Dl-malic acid | 2.923551 | 0.812863 | 0.013939 | 0.043441 |
| 1,2-dimyristoyl-sn-glycero-3-phosphate | 1.51394 | 0.456225 | 0.013948 | 0.043459 |
| Pc(16:1e/17-hdohe) | 2.18017 | 0.762513 | 0.015231 | 0.046716 |
| N-Acetyl-D-Glucosamine 6-Phosphate | 1.107538 | 1.278829 | 0.015569 | 0.047612 |
| 3-dehydroepiandrosterone sulfate | 17.325 | 0.666987 | 0.015723 | 0.048034 |
| 1,2-diarachidonoyl-sn-glycero-3-phosphocholine | 1.060328 | 1.581439 | 0.015895 | 0.048403 |
| 1,2-dipalmitoleoyl-sn-glycero-3-phosphocholine | 2.064513 | 1.344551 | 0.014083 | 0.048412 |
| 5.alpha.-androstan-3.alpha.,17.beta.-diol-o-3-.beta.-glucuronic acid | 3.682724 | 0.651818 | 0.016095 | 0.048892 |

## Table S6. Differential metabolites between IgG4-RP and PC.

| **Name** | **VIP** | **Fold change** | **p-value** | **FDRvalue** |
| --- | --- | --- | --- | --- |
| D-Glutamic acid | 1.2425 | 2.951861 | 3.56E-11 | 1.04E-08 |
| Citrate | 1.933115 | 0.327483 | 8.09E-11 | 2.09E-08 |
| Caftaric acid | 1.173515 | 0.313041 | 8.97E-11 | 2.27E-08 |
| 1-stearoyl-2-myristoyl-sn-glycero-3-phosphocholine | 5.352048 | 3.886345 | 1.02E-10 | 2.44E-08 |
| 1,6-anhydro-2,3-o-isopropylidene-.beta.-d-mannopyranose | 1.732137 | 0.537167 | 3.06E-10 | 4.36E-08 |
| 1-stearoyl-2-palmitoyl-sn-glycero-3-phosphocholine | 1.476231 | 3.323185 | 4.34E-10 | 5.23E-08 |
| 3-hydroxyoctanoic acid | 1.961405 | 0.532652 | 4.93E-10 | 5.67E-08 |
| 1-palmitoyl-2-lauroyl-sn-glycero-3-phosphorylcholine | 12.66857 | 4.112833 | 1.16E-09 | 1.03E-07 |
| 1,2-distearoyl-sn-glycero-3-phospho-(1'-rac-glycerol) | 3.701732 | 2.90181 | 1.87E-09 | 1.5E-07 |
| Fenoxaprop | 2.976351 | 0.494592 | 5.7E-09 | 3.36E-07 |
| 1-stearoyl-2-arachidonoyl-sn-glycero-3-phosphoserine | 5.14044 | 15.63721 | 1.91E-08 | 9.06E-07 |
| N.omega.-hydroxy-nor-l-arginine | 2.26813 | 0.508069 | 1.31E-08 | 1.54E-06 |
| (2-aminoethoxy)[3-[hexadec-1-en-1-yloxy]-2-[icosa-5.8.11.14-tetraenoyloxy]propoxy]phosphinic acid | 5.874508 | 3.3196 | 7.53E-08 | 2.61E-06 |
| Carnitine | 6.996888 | 1.742302 | 7.31E-08 | 5.92E-06 |
| Niacinamide | 3.498839 | 2.669027 | 1.75E-07 | 1.16E-05 |
| Taurine | 8.802295 | 2.357821 | 4.87E-07 | 1.22E-05 |
| [6]-gingerol | 1.799109 | 1.704788 | 6E-07 | 1.46E-05 |
| 5-methyl-5-phenylhydantoin | 1.261191 | 1.833017 | 4.1E-07 | 2.33E-05 |
| L-pyroglutamic acid | 6.361133 | 0.462304 | 1.76E-06 | 3.64E-05 |
| Maltotetraose | 1.551883 | 7.184185 | 3.09E-06 | 5.83E-05 |
| Sarcosine | 1.746077 | 2.051274 | 1.81E-06 | 6.99E-05 |
| Ps 40:4 | 1.159399 | 4.026338 | 6.47E-06 | 0.000109 |
| Pc(18:1e/12-hete) | 1.857269 | 2.497375 | 8.06E-06 | 0.00013 |
| Tartronate | 1.559596 | 1.783621 | 1.06E-05 | 0.000162 |
| 1-oleoyl-2-palmitoyl-sn-glycero-3-phosphocholine | 2.432306 | 2.292612 | 1.61E-05 | 0.000232 |
| 2-hydroxy-6-methylquinoline-3-carbaldehyde | 3.370582 | 0.142124 | 1.83E-05 | 0.000258 |
| Pc(18:1e/9-hode) | 2.990508 | 1.88393 | 1.86E-05 | 0.00026 |
| L-hydroxyarginine | 4.482655 | 1.902815 | 1.04E-05 | 0.000263 |
| Pantoprazole | 1.447087 | 166.7705 | 1.51E-05 | 0.000352 |
| 3',5'-cyclic inosine monophosphate | 1.877388 | 0.75014 | 2.74E-05 | 0.000358 |
| N-(octadecanoyl)sphing-4-enine-1-phosphocholine | 8.85579 | 0.373988 | 2.1E-05 | 0.000456 |
| Pc(18:0e/8-hete) | 2.394232 | 1.917536 | 4.48E-05 | 0.000539 |
| Pe(16:0e/12-hete) | 5.248622 | 1.965372 | 4.61E-05 | 0.000551 |
| Pc(18:1e/14,15-eet) | 2.092152 | 1.935873 | 5.22E-05 | 0.000615 |
| Dl-lactate | 7.621899 | 0.19991 | 6.32E-05 | 0.000728 |
| Linoleoylcarnitine | 3.969208 | 0.57512 | 4.12E-05 | 0.000752 |
| N-tetracosanoyl-4-sphingenyl-1-o-phosphorylcholine | 4.529629 | 0.28961 | 4.39E-05 | 0.000787 |
| Diacetyl | 1.307427 | 2.83655 | 7.83E-05 | 0.001232 |
| Vitamin c | 1.206399 | 0.559201 | 0.000144 | 0.001429 |
| 2-Amino-2-methyl-1,3-propanediol | 3.096159 | 1.790276 | 9.89E-05 | 0.001478 |
| Daunorubicin | 1.078145 | 2.309113 | 0.00012 | 0.001708 |
| Tris(hydroxymethyl)aminomethane | 1.475452 | 0.049932 | 0.000137 | 0.001903 |
| 1,3-benzenediol, 4-chloro-6-[5-[[2-(4-morpholinyl)ethyl]amino]-1,2-benzisoxazol-3-yl]- | 1.478423 | 1.542576 | 0.000151 | 0.002038 |
| Pc(18:1e/8-hepe) | 1.162104 | 2.284457 | 0.000301 | 0.002618 |
| N-alpha-acetyl-l-lysine | 2.889349 | 1.665302 | 0.000212 | 0.00264 |
| Chenodeoxycholate | 2.912469 | 1.793328 | 0.000238 | 0.002858 |
| 1,2-dilauroyl-sn-glycero-3-phosphatidylcholine | 5.25443 | 0.618965 | 0.000249 | 0.002965 |
| Val-Ala-Lys | 1.676804 | 1.70203 | 0.000269 | 0.003138 |
| L-Glutamine | 1.095059 | 3.355583 | 0.00032 | 0.003585 |
| 5-aminovaleric acid betaine | 1.187632 | 0.38414 | 0.000321 | 0.003588 |
| Cis,cis-muconic acid | 18.69659 | 0.739049 | 0.000469 | 0.003754 |
| Hydrocortisone | 1.645863 | 0.502234 | 0.00036 | 0.003911 |
| 1-o-hexadecyl-2-o-(2e-butenoyl)-sn-glyceryl-3-phosphocholine | 2.302361 | 1.401077 | 0.000377 | 0.004025 |
| Tuberostemonine | 6.640428 | 1.676731 | 0.000405 | 0.004242 |
| Pyruvaldehyde | 3.213026 | 0.059145 | 0.000597 | 0.004559 |
| Di(2,6-dimethyl-4-heptyl) phthalate | 1.243748 | 1.496743 | 0.00046 | 0.004683 |
| Ser-Thr | 1.592281 | 1.66442 | 0.000536 | 0.00527 |
| Pc 42:10 | 1.984388 | 3.909569 | 0.000759 | 0.005515 |
| Caylin-1 | 1.891224 | 0.757799 | 0.000609 | 0.005803 |
| Inosine | 1.118675 | 0.381218 | 0.000617 | 0.005851 |
| 1',3'-bis[1,2-dilinoleoyl-sn-glycero-3-phospho]-sn-glycerol | 3.359977 | 2.617097 | 0.000821 | 0.005868 |
| 1-o-hexadecyl-2-o-butanoyl-sn-glyceryl-3-phosphocholine | 1.665059 | 1.523036 | 0.000652 | 0.0061 |
| 2-arachidonoyl-1-palmitoyl-sn-glycero-3-phosphoethanolamine | 6.035932 | 1.926433 | 0.000923 | 0.006457 |
| L-gulono-1,4-lactone | 1.890553 | 1.762256 | 0.000965 | 0.006698 |
| (2-aminoethoxy)[2-[docosa-4.7.10.13.16.19-hexaenoyloxy]-3-[hexadec-1-en-1-yloxy]propoxy]phosphinic acid | 6.487873 | 1.937243 | 0.000993 | 0.006845 |
| Hydroxyisocaproic acid | 1.433458 | 0.428917 | 0.001114 | 0.007496 |
| Sm d34:1 | 1.429529 | 1.680049 | 0.001258 | 0.008279 |
| 17,20-dimethylprostaglandin f1.alpha. | 1.227468 | 0.574952 | 0.001906 | 0.01155 |
| Palmitamide | 2.73962 | 1.414104 | 0.001487 | 0.011561 |
| Lactose | 1.040011 | 2.365021 | 0.001503 | 0.011662 |
| Stearidonic acid | 1.505982 | 0.571364 | 0.001607 | 0.012196 |
| Uridine | 2.74389 | 0.674703 | 0.002053 | 0.012264 |
| alpha-Linolenic acid | 1.128698 | 0.689809 | 0.001772 | 0.013076 |
| Cefadroxil | 1.313087 | 1.865753 | 0.00232 | 0.013553 |
| Dimethylformamide | 1.417442 | 2.28714 | 0.002029 | 0.014501 |
| Creatine | 2.085871 | 3.595447 | 0.002076 | 0.0147 |
| Pc(16:0e/13-hode) | 2.860891 | 1.708619 | 0.002696 | 0.015249 |
| Hypoxanthine | 2.842459 | 0.379984 | 0.002194 | 0.015382 |
| Pe 38:4 | 13.57112 | 1.788485 | 0.002892 | 0.016105 |
| L-Isoleucine | 2.371412 | 1.594932 | 0.002564 | 0.017441 |
| Vecuronium | 1.449688 | 0.58462 | 0.002603 | 0.017641 |
| 1,2-diarachidonoyl-sn-glycero-3-phosphocholine | 1.152149 | 2.245125 | 0.003687 | 0.019629 |
| (+)-.gamma.-tocopherol | 1.38189 | 2.11779 | 0.0042 | 0.021823 |
| S-Methyl-5'-thioadenosine | 1.209444 | 1.949775 | 0.00394 | 0.024191 |
| Tegaserod | 1.435529 | 2.290392 | 0.004088 | 0.024826 |
| Guanidinopropionic acid | 2.707275 | 0.712325 | 0.005022 | 0.025158 |
| 1-pentadecanoyl-sn-glycero-3-phosphocholine | 2.40152 | 1.330924 | 0.004191 | 0.025233 |
| D-glucono-1,5-lactone | 1.556665 | 2.178223 | 0.005744 | 0.0278 |
| L-Pipecolic acid | 1.791426 | 1.657565 | 0.004966 | 0.028822 |
| N,n-dimethylformamide | 1.413924 | 1.990492 | 0.005134 | 0.029526 |
| Palmitoyl sphingomyelin | 20.90629 | 0.695381 | 0.00523 | 0.029988 |
| 1-myristoyl-sn-glycero-3-phosphocholine | 2.867946 | 1.767282 | 0.00563 | 0.031433 |
| Rac-7-hydroxypropranolol | 1.419184 | 13.2999 | 0.005843 | 0.032314 |
| m-Chlorohippuric acid | 2.270353 | 1.482727 | 0.007675 | 0.035013 |
| 1-hexadecyl-sn-glycero-3-phosphocholine | 2.100023 | 1.341452 | 0.006883 | 0.036748 |
| Sphingomyelin (d18:1/18:0) | 2.352898 | 2.753215 | 0.007495 | 0.038851 |
| Alanine | 1.534612 | 2.069957 | 0.007513 | 0.038902 |
| Pe 40:4 | 4.420847 | 1.617764 | 0.009046 | 0.039716 |
| L-threonate | 1.078271 | 1.520994 | 0.009551 | 0.041434 |
| Oleic acid | 1.074095 | 1.510683 | 0.011364 | 0.047101 |
| D-Mannose | 1.19674 | 1.455887 | 0.009793 | 0.047208 |
| N-.alpha.-acetyl-l-ornithine | 3.087918 | 1.513665 | 0.010042 | 0.048036 |
| D-gluconate | 1.079009 | 1.608679 | 0.012153 | 0.04969 |

## Table S7. Differential metabolites between IgG4-RSD and SS.

| **Name** | **VIP** | **Fold change** | **p-value** | **FDRvalue** |
| --- | --- | --- | --- | --- |
| Hydroxyproline | 1.734561 | 1.930616 | 3.39E-15 | 2.26E-12 |
| 1,2-dilauroyl-sn-glycero-3-phosphatidylcholine | 7.143517 | 0.481657 | 3.87E-14 | 2.04E-11 |
| N-tetracosanoyl-4-sphingenyl-1-o-phosphorylcholine | 5.30672 | 0.182547 | 4.18E-13 | 1.39E-10 |
| 2-(5-oxovaleryl)phosphatidylcholine | 1.123393 | 5.555362 | 1.05E-12 | 2.73E-10 |
| N-(octadecanoyl)sphing-4-enine-1-phosphocholine | 8.699839 | 0.392633 | 2.59E-10 | 2.5E-08 |
| Palmitoyl sphingomyelin | 29.11013 | 0.57046 | 2.92E-10 | 2.74E-08 |
| Lpc 16:0 | 33.8211 | 1.815279 | 9.34E-10 | 7.17E-08 |
| Imidazoleacetic acid | 1.178872 | 4.073081 | 1.22E-09 | 8.92E-08 |
| Tuberostemonine | 5.469161 | 1.801041 | 1.59E-09 | 1.12E-07 |
| Chenodeoxycholate | 2.10281 | 1.72322 | 3.51E-09 | 2.29E-07 |
| Omeprazole sulfone n-oxide | 1.522853 | 81.95712 | 7.03E-08 | 2.73E-06 |
| L-hydroxyarginine | 3.394925 | 1.890611 | 1.78E-07 | 5.79E-06 |
| N-acetylglutamine | 2.191502 | 0.329298 | 3.93E-09 | 6.09E-06 |
| Stearoyl-l-carnitine | 1.316617 | 1.619889 | 2.2E-07 | 6.84E-06 |
| N-acetyltryptophan | 1.304002 | 0.434847 | 1.03E-08 | 9.68E-06 |
| Glycerophosphocholine | 2.719785 | 1.418818 | 4.49E-07 | 1.22E-05 |
| N-acetyldihydrosphingosine | 1.257039 | 0.333382 | 5E-07 | 1.34E-05 |
| L-palmitoylcarnitine | 1.917162 | 1.497602 | 9.58E-07 | 2.23E-05 |
| Niacinamide | 2.971789 | 2.615264 | 1.43E-06 | 3.05E-05 |
| N-acetylsphingosine | 1.257766 | 0.292391 | 1.8E-06 | 3.68E-05 |
| Glutamic acid | 1.342055 | 1.884074 | 1.23E-07 | 3.74E-05 |
| m-Chlorohippuric acid | 4.239988 | 0.598302 | 1.85E-07 | 4.38E-05 |
| Thr-Gly | 1.040878 | 1.221191 | 2.27E-07 | 4.94E-05 |
| Creatinine | 10.38491 | 1.712931 | 4.54E-06 | 7.78E-05 |
| 2-Amino-2-methyl-1,3-propanediol | 2.15303 | 1.708394 | 5.5E-06 | 9.11E-05 |
| Uracil | 1.802637 | 1.663396 | 8.04E-06 | 0.000124 |
| 1-o-hexadecyl-2-o-(2e-butenoyl)-sn-glyceryl-3-phosphocholine | 1.6583 | 1.349099 | 1.37E-05 | 0.000194 |
| N-.alpha.-acetyl-l-ornithine | 3.125632 | 2.057231 | 1.79E-05 | 0.000241 |
| 1-(1z-octadecenyl)-2-(5z,8z,11z,14z-eicosatetraenoyl)-sn-glycero-3-phosphoethanolamine | 2.058616 | 0.443573 | 2.45E-05 | 0.000315 |
| Linoleoylcarnitine | 2.698172 | 1.702714 | 3.4E-05 | 0.00041 |
| 1,2-diamino-2-methylpropane | 1.363265 | 1.354966 | 3.59E-05 | 0.00043 |
| 1',3'-bis[1,2-dilinoleoyl-sn-glycero-3-phospho]-sn-glycerol | 4.367418 | 2.546485 | 6.29E-06 | 0.000437 |
| 1-stearoyl-2-myristoyl-sn-glycero-3-phosphocholine | 6.051186 | 2.05572 | 7.23E-06 | 0.000479 |
| Caylin-1 | 1.454769 | 0.879482 | 5.26E-05 | 0.000577 |
| Cholesteryl sulfate | 14.82519 | 1.917815 | 1.24E-05 | 0.000682 |
| 1,2-distearoyl-sn-glycero-3-phospho-(1'-rac-glycerol) | 4.175716 | 1.721601 | 1.69E-05 | 0.000856 |
| Nocardamine | 1.558048 | 0.572971 | 1.84E-05 | 0.000905 |
| Cis-13-docosenoic acid | 1.588903 | 0.648507 | 0.000109 | 0.001058 |
| 1-(1z-hexadecenyl)-sn-glycero-3-phosphocholine | 2.401929 | 1.297535 | 0.000118 | 0.00113 |
| Di(2,6-dimethyl-4-heptyl) phthalate | 1.237313 | 1.712773 | 0.000139 | 0.001298 |
| Taurocholate | 1.658581 | 0.25685 | 3.77E-05 | 0.001459 |
| 1-stearoyl-2-arachidonoyl-sn-glycero-3-phosphoserine | 6.555118 | 3.314045 | 3.74E-05 | 0.001459 |
| Pristimerin | 1.109294 | 1.59357 | 3.79E-05 | 0.001462 |
| Dimethyl .mu.-truxinate | 1.026547 | 0.043128 | 0.000162 | 0.001473 |
| Erucamide | 8.919168 | 0.626766 | 0.000176 | 0.001567 |
| Pi 38:5 | 9.973306 | 2.7175 | 5.1E-05 | 0.001778 |
| Lpc 18:2 | 16.02302 | 1.320598 | 0.000237 | 0.001988 |
| D-erythro-sphingosine-1-phosphate | 1.293383 | 1.557662 | 6.49E-05 | 0.002076 |
| 1-stearoyl-2-oleoyl-sn-glycero-3-phosphocholine | 1.059744 | 1.341842 | 6.91E-05 | 0.002151 |
| Choline | 2.707529 | 1.959025 | 0.00032 | 0.002551 |
| (+)-6-aminopenicillanic acid | 3.450874 | 1.352823 | 9.15E-05 | 0.002557 |
| Pi(18:0/8,9-eet) | 2.480829 | 2.178003 | 9.64E-05 | 0.002646 |
| Acetylcarnitine | 7.14569 | 1.615743 | 0.000352 | 0.002749 |
| Pi(18:0/9-hode) | 1.287161 | 2.025946 | 0.00011 | 0.002876 |
| Pc(18:1e/8-hepe) | 1.718268 | 2.027072 | 0.000114 | 0.002943 |
| N-stearoyltaurine | 1.018491 | 1.532645 | 0.000117 | 0.002995 |
| Ps 40:4 | 1.369014 | 2.344885 | 0.000139 | 0.003396 |
| 2-methylbutyryl-l-carnitine | 1.126342 | 1.754248 | 0.000482 | 0.003545 |
| Dl-a-hydroxybutyric acid | 2.741314 | 1.840895 | 0.000155 | 0.003654 |
| 1-octadecanoyl-2-octadecenoyl-sn-glycero-3-phosphocholine | 13.03827 | 0.692788 | 0.00051 | 0.0037 |
| L-propionylcarnitine | 1.972347 | 1.756365 | 0.000538 | 0.003851 |
| 1-hexadecyl-2-(9z-octadecenoyl)-sn-glycero-3-phosphoethanolamine | 1.141317 | 1.456189 | 0.000174 | 0.003901 |
| Betaine | 8.441935 | 0.765142 | 0.000662 | 0.004597 |
| Diacetyl | 1.075147 | 2.919396 | 0.00077 | 0.005236 |
| Carnitine | 2.233436 | 1.113362 | 0.000823 | 0.005501 |
| 1-docosahexaenoyl-2-stearoyl-sn-glycero-3-phosphocholine | 6.312054 | 0.623692 | 0.00085 | 0.005643 |
| Maltotetraose | 1.71463 | 2.668634 | 0.000301 | 0.005649 |
| Pc(18:1e/12-hete) | 2.030058 | 1.767478 | 0.000307 | 0.005697 |
| Pi 40:8 | 1.304389 | 2.69681 | 0.000311 | 0.005755 |
| Beta-octylglucoside | 1.840841 | 1.735367 | 0.000871 | 0.005755 |
| N.omega.-hydroxy-nor-l-arginine | 1.079234 | 0.815767 | 0.000876 | 0.00578 |
| Pi 38:4 | 19.35025 | 1.526875 | 0.000432 | 0.007024 |
| 1-(1z-octadecenyl)-2-(5z,8z,11z,14z-eicosatetraenoyl)-sn-glycero-3-phosphocholine | 2.733263 | 0.74581 | 0.001149 | 0.007154 |
| 1-stearoyl-2-palmitoyl-sn-glycero-3-phosphocholine | 1.525145 | 2.039595 | 0.000458 | 0.007291 |
| 1-monolinoleoyl-rac-glycerol | 1.531145 | 0.646868 | 0.001361 | 0.008198 |
| 1-Palmitoylglycerol | 3.420355 | 0.683632 | 0.001376 | 0.008273 |
| Aleuritic acid | 1.216642 | 1.218574 | 0.00056 | 0.008398 |
| EDTA | 1.130047 | 1.953133 | 0.001426 | 0.00852 |
| Fenazaflor | 1.564519 | 0.86276 | 0.001451 | 0.00864 |
| 1-oleoyl-2-palmitoyl-sn-glycero-3-phosphocholine | 2.524622 | 1.610683 | 0.000641 | 0.009268 |
| 2-linoleoylglycerol | 4.967872 | 0.560679 | 0.001739 | 0.010024 |
| 1-hexadecyl-sn-glycero-3-phosphocholine | 2.252885 | 1.738112 | 0.001766 | 0.010162 |
| 1,2-diarachidonoyl-sn-glycero-3-phosphocholine | 1.076405 | 1.731261 | 0.000829 | 0.011111 |
| 4-amino-n-(tert-butyl)benzamide | 1.0084 | 0.413426 | 0.002196 | 0.01211 |
| N-palmitoyltaurine | 1.016216 | 1.427129 | 0.000962 | 0.012263 |
| 1-hexadecyl-2-(9z-octadecenoyl)-sn-glycero-3-phosphocholine | 2.058325 | 0.649347 | 0.002253 | 0.01238 |
| 1-heptadecanoyl-sn-glycero-3-phosphocholine | 2.744859 | 1.563294 | 0.002593 | 0.013945 |
| 1-palmitoyl-2-lauroyl-sn-glycero-3-phosphorylcholine | 12.03973 | 1.551167 | 0.001175 | 0.01423 |
| (2s,3r,5r,10r,13r,14s,17s)-2,3,14-trihydroxy-10,13-dimethyl-17-[(2r,3r,5r)-2,3,6-trihydroxy-5,6-dimethylheptan-2-yl]-2,3,4,5,9,11,12,15,16,17-decahydro-1h-cyclopenta[a]phenanthren-6-one | 2.837854 | 1.699046 | 0.001236 | 0.014728 |
| (e,2s,3r,4r,5s)-4-acetyloxy-2-amino-3,5,14-trihydroxyicos-6-enoic acid | 1.262059 | 0.639407 | 0.001276 | 0.015053 |
| 2-ketohexanoic acid | 15.02518 | 1.443188 | 0.001287 | 0.015113 |
| 2-sdahma [dmed-fahfa] | 1.495538 | 0.566242 | 0.003127 | 0.016201 |
| Isomaltose | 1.13355 | 2.542024 | 0.001444 | 0.016361 |
| Dl-lactate | 3.708342 | 1.749585 | 0.001496 | 0.016712 |
| 15-ketoiloprost | 4.87873 | 1.255666 | 0.001614 | 0.017564 |
| Cefadroxil | 1.193571 | 0.733398 | 0.001706 | 0.018131 |
| Rhamnetin | 3.05755 | 0.772266 | 0.002 | 0.0203 |
| Salicylic acid .beta.-d-o-glucuronide | 1.016124 | 1.129855 | 0.002005 | 0.0203 |
| N6-(1-iminoethyl)-l-lysine | 1.224766 | 1.525675 | 0.004955 | 0.023136 |
| (2-aminoethoxy)[3-[hexadec-1-en-1-yloxy]-2-[icosa-5.8.11.14-tetraenoyloxy]propoxy]phosphinic acid | 5.781667 | 1.475003 | 0.002677 | 0.02504 |
| 1,2-dioleoyl-sn-glycero-3-phosphoethanolamine-n,n-dimethyl | 3.99334 | 1.491047 | 0.00278 | 0.025792 |
| Urapidil | 1.304697 | 1.623712 | 0.002821 | 0.026038 |
| Citrate | 1.116475 | 0.723831 | 0.00315 | 0.028149 |
| 1-Stearoyl-2-arachidonoyl-sn-glycerol | 5.406463 | 0.466135 | 0.006594 | 0.028962 |
| Rac-7-hydroxypropranolol | 1.587202 | 13.62076 | 0.006895 | 0.029993 |
| Irbesartan | 3.606282 | 2.054244 | 0.003702 | 0.0313 |
| 1-stearoyl-2-linoleoyl-sn-glycero-3-phosphoethanolamine | 10.05028 | 1.195848 | 0.003978 | 0.032804 |
| Ornithine | 1.65754 | 1.786492 | 0.008337 | 0.034451 |
| 1-pentadecanoyl-sn-glycero-3-phosphocholine | 1.280643 | 1.23599 | 0.008398 | 0.034629 |
| Pe(16:0e/10-hdohe) | 1.023618 | 1.485438 | 0.004354 | 0.034922 |
| Fenoxaprop | 1.787502 | 0.837566 | 0.004717 | 0.037043 |
| Perifosine | 1.18303 | 1.535003 | 0.004741 | 0.037116 |
| Pe 38:4 | 11.3491 | 1.3649 | 0.004934 | 0.037949 |
| Hydroquinidine | 4.01421 | 1.226499 | 0.005125 | 0.038797 |
| 1,2-dioleoyl-sn-glycero-3-phospho-l-serine | 1.017179 | 1.916935 | 0.005169 | 0.038913 |
| D-gluconate | 1.040865 | 0.752806 | 0.005334 | 0.039818 |
| Thymol-beta-d-glucoside | 3.961091 | 1.23176 | 0.005615 | 0.04132 |
| Pi(16:0e/15-hete) | 3.132251 | 1.730077 | 0.005774 | 0.04204 |
| 2-hydroxy-6-methylquinoline-3-carbaldehyde | 1.376699 | 2.19669 | 0.005854 | 0.042446 |
| .beta.-amyrin acetate | 1.15648 | 1.328158 | 0.011821 | 0.045059 |
| Taurine | 7.531916 | 1.499356 | 0.007312 | 0.049279 |

## Table S8. Clinical features of relapsed and non-relapsed IgG4-RD.

|  | **relapsed IgG4-RD**  **(n=30)** | **non-relapsed IgG4-RD (n=30)** |
| --- | --- | --- |
| **Demography**  Sex (male，%) | 21 (70.0%) | 22 (73.3%) |
| Age at onset (years, mean ± SD) | 53.86±11.96 | 53.17±12.93 |
| Disease duration (months, median, IQR) | 24(8-60) | 36(5-54) |
| Affected organ numbers (mean ± SD) | 3.62±1.35 | 3.55±1.45 |
| IgG4 (g/L) | 13.30 (7.54-22.40) | 12.30 (6.04-21.05) |
| **Organ involvement (n, %)** |  |  |
| Lacrimal gland | 19 (63.3%) | 20 (66.7%) |
| Parotid gland | 5 (16.7%) | 4 (13.3%) |
| Submandibular gland | 23 (76.7%) | 20 (66.7%) |
| Pancreas | 10 (33.3%) | 9 (30.0%) |
| Bile duct | 6 (20.0%) | 4 (13.3%) |
| Lung | 8 (26.7%) | 10 (33.3%) |
| Kidney | 3 (10.0%) | 2 (6.7%) |
| Retroperitoneum | 4 (13.3%) | 4 (13.3%) |
| Paranasal sinus | 11 (36.7%) | 13 (43.3%) |
| Thyroid | 0 | 0 |
| Pituitary | 0 | 0 |
| Lymph node | 15 (50.0%) | 10 (33.3%) |

**Variables are described as mean ± S.D. or median, IQR according to whether they satisfied normal distribution.**
